# Concreteness and emotional valence of episodic future thinking (EFT) independently affect the dynamics of intertemporal decisions

**DOI:** 10.1371/journal.pone.0217224

**Published:** 2019-05-28

**Authors:** Cinzia Calluso, Annalisa Tosoni, Loreta Cannito, Giorgia Committeri

**Affiliations:** 1 Department of Neuroscience, Imaging and Clinical Sciences, Gabriele d’Annunzio University, and Institute for Advanced Biomedical Technologies (ITAB), Chieti Scalo, Italy; 2 Department of Business and Management, LUISS Guido Carli University, Rome, Italy; University of Bologna, ITALY

## Abstract

During intertemporal decisions, the value of future rewards decreases as a function of the delay of its receipt (temporal discounting, TD). Since high discount rates have been associated with a series of problematic behaviours and clinical conditions, current research has focused on possible modulators of TD. Specifically, a reduction of individual discount rates has been shown during episodic future thinking (EFT), wherein time intervals are anchored to personal future events. However, it is not entirely clear whether this effect is mediated by a change in the representation of future events (i.e., from abstract to concrete) or by a positive-emotion modulation. Here, we investigated this issue by manipulating the valence of the EFT (i.e., using negative, neutral and positive episodic tags), and by collecting explicit and implicit measures of behaviour. The results showed a significant reduction of TD in all the three emotional conditions compared to the baseline, with differences among them, thus suggesting the existence of a cumulative effect of the concreteness and affective components of the EFT. The analyses of implicit measures additionally revealed that this effect was mediated by a simultaneous increase/decrease of attraction toward the delayed/immediate alternative. Finally, these effects appeared to be modulated by participants’ baseline discounting preferences. These findings provide important insights on clinical applications in reward-related disorders.

## Introduction

The preference for smaller sooner rewards over larger delayed ones, known as temporal discounting, is not only a commonly observed behaviour during many daily-life situations but also the predominant choice pattern of a series of sub-optimal behaviours and clinical conditions [1–4]. Prior research indeed has demonstrated that steep delay discounting predicts initiation of smoking in adolescence [1,5,6], and that individual differences in intertemporal choice tasks are associated with dysfunctional behaviours (including alcoholism [7,8], drug abuse [9], pathological gambling [10,11], credit card borrowing [12]) and life-style habits [13].

But why do people discount future rewards? According to recent proposals, intertemporal choice is a complex behaviour that can be explained on the basis of a dynamic interplay between three neurocognitive systems [14,15]: i) a valuation network responsible of the computation of subjective value of future rewards [16–20]; ii) a self-control network responsible of the ability to delay gratifications through cognitive control and conflict monitoring [21–24]; iii) a prospective memory network responsible of the representation of future outcomes [25–27].

This latter component, in particular, has received increasing attention in the last few years because it has been shown that it can be modulated by a series of contextual factors associated with a significant reduction of the discount rates [2,28]. For example, it has been shown that anchoring time intervals to subject-specific real-life events [i.e., episodic tagging, or episodic future thinking (EFT) manipulation] significantly reduces participants’ tendency to discount future rewards, and this effect has been interpreted in terms of an alteration of the associated time construal level (i.e., a shift in the cognitive representation of time from high-level to low-level) [29,30]. In particular, it has been proposed that the effect of enhanced farsightedness by the EFT manipulation is mediated by a shift in the representation of future events from an abstract to a concrete level, resulting in an increased perception of temporal proximity of the same events to the present and a consequential increase of their selection. From a neural perspective, this effect appears to be linked to the functional coupling between the valuation network and the prospective memory network [25].

Importantly, the effect of increased farsightedness during the EFT manipulation has been observed in many different studies, thus appearing to represent a powerful modulator of intertemporal choice behaviour [31–34]. Nevertheless, because in these studies individuals only imagined positive future events, it has been argued that the effect may be driven by the positive emotional valence of the episodic tags rather than by a modulation of the time construal level [28]. Hence, to disentangle the specific contribution of these two factors, some studies have introduced a manipulation of the EFT emotional valence. The results obtained, however, still appear inconclusive. Liu and colleagues (2013), for example, have found that while positive episodic tags reduced the preference for immediate rewards, negative tags increased it and neutral ones did not produce any effect, thus suggesting that emotional valence is the main determinant of the EFT effect [35]. The same results were also replicated by a recent study by Zhang and colleagues (2018) [36]. Conversely, in the study by Lin and Epstein (2014) it was shown that both neutral and positive episodic tags were associated with a reduction of discount rates, hence supporting the hypothesis of a concreteness effect [37]. Furthermore, Bulley and colleagues (2019) have recently observed a significant reduction of the discount rates during both positive and negative tags compared to neutral imagery [38]. Therefore, based on the currently available literature, it is still not entirely clear whether the effect of enhanced farsightedness during episodic future thinking is mediated by a change in the representation of future events (i.e., from an abstract to a concrete level) or by a positive-emotion modulation.

In this respect, however, it is important to note that these studies were conducted using between-subjects’ designs [e.g., positive valence to one group of subjects and neutral (or negative) valence to a different group], which might have reduced the probability to observe significant differences given the well-known large inter-subject variability of discount rates.

Here we sought to systematically investigate the specific contribution of concreteness and emotional valence of an EFT manipulation within the same group of subjects. To this aim, we collected intertemporal decisions from 65 healthy participants during a baseline session and an EFT session in which we manipulated the emotional valence (i.e., negative, neutral or positive) of the episodic tags (i.e., future events of the participants’ personal life). Importantly, to examine the effect of EFT emotional valence and concreteness on decision formation, not only the choice outcome was recorded in each trial, but also the kinematics of the mouse movements during the selection process. The latter measure, indeed, is currently considered an important measure of the cognitive processing underlying decision formation. In particular, by studying the temporal evolution and the extent to which the response movements are pulled toward each of the two choice alternatives, the analysis of kinematic data provides relevant information about the unfolding decision with a fine-grained spatial and temporal resolution [39–41]. With specific reference to discounting behaviour, moreover, we have previously shown that individual differences in discounting preferences (i.e., short-sighted vs. farsighted) were reliably reflected in different kinematics patterns and that these patterns were indicative of the cognitive mechanisms underlying the intertemporal decision [42]. In particular, while farsighted participants exhibited a clear bias toward the delayed option, which was evident during both the selection of the delayed option (i.e., straight trajectories), as well as during the selection of the immediate option (i.e., with abrupt change of direction only at the very end of the selection process), a similar curvature for the two choice alternatives was observed in the choice trajectories of discounters’ individuals, which suggested a parallel choice competition process.

Along the same lines, here we examined the cognitive mechanisms underlying the manipulation of emotional valence of episodic future thinking on discount rates. In order to highlight potential dissociations between subjects with different discounting preferences, this was done by analysing the changes in both the explicit choice’ preferences and in the implicit kinematic patterns of attraction/uncertainty toward the two options during the selection process.

## Methods

### Subjects

Sixty-five right-handed healthy volunteers (24 males, mean age: 23.66 ± 3.08) participated in the study after providing written informed consent in accordance with the ethical standards of the 1964 Declaration of Helsinki and the study approval by the Ethics Committee of the “G. d’Annunzio” University.

Ten participants were excluded from the analysis because they showed no choice variability in the intertemporal task (they gave only “now” or “later” responses in all the trials of at least one block: baseline, negative, neutral or positive) which precluded the estimation of the individual discount rates and the statistical testing. Hence, the final sample included fifty-five experimental subjects (21 males, mean age: 23.87 ± 3.21). The sample size was calculated based on a power analysis [G*Power 3.1.9.2 (http://www.gpower.hhu.de/en.html) [43,44]] conducted on the effect size (Cohen’s d, values varying between 0.53 and 0.67) reported in a previous study on the same topic [35]. This analysis indicated a required sample size ranging between 49 and 64 participants. We also found 55 to be a critical number of participants, since the estimated power remained stable above this number.

### Behavioural paradigm

**Fig 1:**
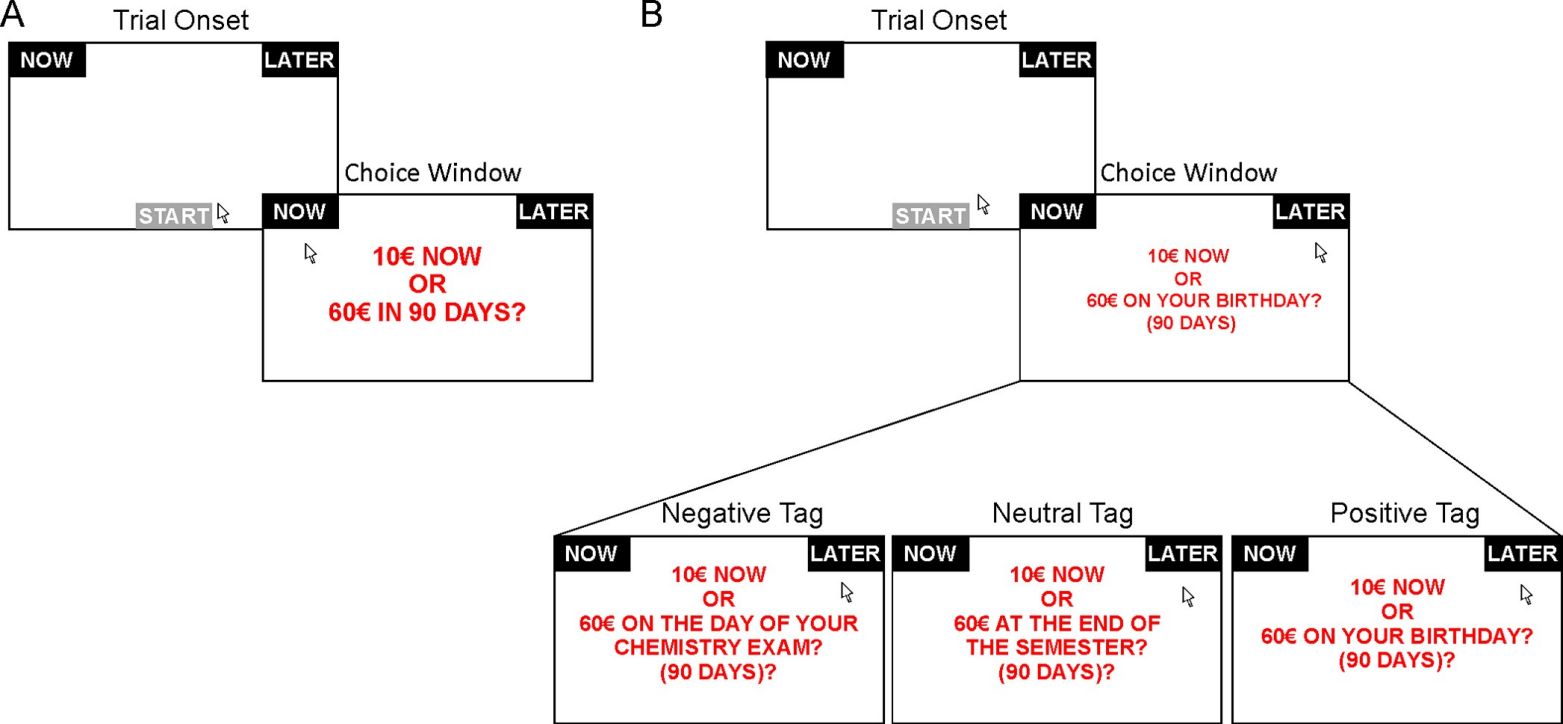
Behavioural paradigm. (A) Baseline session: to ensure a proper recording of the mouse position at the beginning of each trial participants were instructed to press the “START” button positioned at the central bottom of the screen to visualize the choice options. Participants were subsequently instructed to express their preference by clicking on the corresponding response button. (B) Episodic future thinking session (EFT): as in the baseline session participants were firstly instructed to press the “START” button to visualize the choice options. In this session, however, along with the amount and delay time, an additional subject-specific episodic cue was displayed. The session was divided into three experimental blocks–with negative, neutral and positive emotional valences cues—presented in a counterbalanced order across participants.

During the baseline intertemporal choice task, participants were asked to make a series of hypothetical (i.e., subjects did not receive any payment for their participation in the study) choices between an immediate fixed amount of money (i.e., 10€) and a delayed one. The latter option was parametrically manipulated across seven amounts (i.e., 15€, 25€, 30€, 40€, 45€, 55€ and 60€) and six waiting times (i.e., 7, 15, 30, 60, 90, and 180 days), thus obtaining 42 different choice pairs (see [42,45,46] for a similar task design; Fig 1A). Each choice pair was repeated five times for each condition, thus resulting in a total of 210 trials. Following task execution, a questionnaire was administered requiring participants to provide a series of real future events with different emotional valences that were supposed to happen at the time of the delayed reward delivery (7, 15, 30, 60, 90 and 180 days after the second behavioural session, i.e. the EFT session, see below). More specifically, participants were instructed to imagine, as vividly as possible, and to list a series of personal events with a positive, negative or neutral emotional valence that were likely to happen around those time intervals. Upon questionnaire completion, participants were asked to rate the provided events on a 5-point Likert scale (from 0 to 4) according to their personal relevance, arousal and valence. The personal events were used as episodic tags attached to the delayed options during the EFT session, which was collected three days after the baseline session and included a total of 630 trials [i.e., 210 x 3 (negative, neutral, and positive conditions)].

Although an analysis conducted on the rating scores confirmed the prediction that positive events were normally associated with a higher level of both arousal and relevance [significant effect of emotional valence on both arousal (F_2, 128_ = 103.77, p < 0.001) and relevance rating (F_2, 128_ = 113.14, p < 0.001) in a repeated measures ANOVA with emotional valence as within-subject factor], the events presented in the EFT session were roughly matched for relevance (negative: m = 3.13 ± 0.12 s.d.; neutral: m = 2.15 ± 0.13 s.d.; positive: m = 3.41 ± 0.09 s.d.) and arousal (negative: m = 2.68 ± 0.14 s.d.; neutral: m = 1.80 ± 0.13 s.d.; positive: m = 3.17 ± 0.12 s.d.) across the three emotional valence conditions.

During the EFT session, participants were instructed to perform the same intertemporal choice task as in the baseline session. As indicated above, however, the delayed option was tagged with the personal events provided by the participants during the baseline session (see Fig 1B). Notably, no explicit instruction was given to participants about mental imagination or memory reactivation of the episodic tags and they were only prompted to consider the tags in order to learn at which point in time the reward was going to become available. The EFT session was divided into three different blocks, each involving episodic cues of a specific emotional valence (i.e., negative, neutral and positive). These blocks were presented in a counterbalanced order across participants.

Both the baseline and the EFT session were run using MouseTracker Software [47] in order to record the x-y position of the mouse cursor during the entire experiment. To ensure a proper recording of mouse movements, at the beginning of each trial, participants were instructed to click on a *start* button, positioned at the bottom centre of the screen. Then, subjects visualized the choice options and were instructed to select the preferred one by clicking on the corresponding response button (“*now*” vs. “*later*”). The buttons were positioned at the top left and top right corner of the screen, equidistantly from the *start* button. The association between the spatial position and the choice option was counterbalanced across participants. There was no overall time limit imposed to the participants’ choices, but in line with the recommendations provided for the collection of kinematic data and the methodology employed in previous kinematic studies [40,42,47–53], in the case that no movement was recorded within 2 seconds following the start of the trial a pop-out window was shown, inviting participants to initiate the movement and to more generally speed up their decision time. This had the function of minimizing the possibility that the response movement was initiated only once the decision process had been completely finalized, and therefore, to maximize the “on-line” properties of the recorded measure [40,47,53].

### Discount rate estimation

Using the subjects’ observed behaviour and the well-known hyperbolic function as a model to describe the decline of subjective value with increasing time delays [54,55], we estimated the subject-specific discount rates (*k* parameter), in line with a standard routine also employed in a series of previous studies (see [16,18,42,45,46,56]). As a first step, we calculated–for each time delay—the fraction of times in which participants selected the future reward over the immediate one as a function of the objective amount of the delayed option. Then, the points of subjective equivalence (*pse*)–defined as the amount at which participant would choose the immediate and the delayed reward with equal frequency—were estimated by fitting these data with a logistic function implemented in MATLAB. Subjective values (*SV*) for each time delay were thus calculated as the ratio between the immediate amount (10€) and the *pse*, using the following equation:

$$SV=\frac{10}{pse}$$ (1)

where 10 was the amount of money that was immediately available (i.e., 10€). This procedure ensured that subjective values were normalized to the immediate amount of money.

Finally, subject-specific discount rates (*k*) were estimated by fitting *SV* and time delays (*D*) with the hyperbolic function [54,55]:

$$SV=\frac{1}{\left(1+kD\right)}$$ (2)

the goodness-of-fit between the hyperbolic model and the data was overall high: R^2^ ~ 0.80 (baseline: R^2^ = ~0.82; negative: R^2^ = ~0.79; neutral: R^2^ = ~0.80; positive: R^2^ = ~0.78). A logarithmic transformation, prior to statistical testing, was performed to account for skewed distribution (Kolmogorov-Smirnov test of normality: p > 0.20).

### Spatial and temporal measures of mouse kinematics

The position of the mouse cursor (i.e., x- and y-coordinate pairs) was continuously recorded during the entire experiment at a 70 Hz sample rate. Because each choice has a different length, and consequently, a different number of coordinate pairs, we conducted a normalized-time analysis. As a first step, trajectories were resampled in a definite number of time steps (n = 101), by performing a linear interpolation. Then, the normalized trajectories were used to extrapolate two spatial measures: the *maximum deviation* (MD) and the *area under the curve* (AUC). Thus, a theoretical trajectory–namely, the straight line connecting trajectories’ start and endpoints—was computed and the MD was calculated as the maximum perpendicular deviation between the real and the theoretical trajectories, while the AUC was calculated as the geometric area between them [47,57]. The *x-flip* was also included as spatial measure and was defined as the number of times the mouse cursor changes direction along the x-axis (i.e., decision axis in the current paradigm).

A series of temporal measures were also collected during the experiment, including the *total time*, defined as the time between the onset of the trial and the selection of the response (i.e., response time); the *initiation time*, defined as the latency between the trial onset and the movement initiation, and the *motion time*, defined as the time between the movement initiation and the response selection.

In order to avoid excessive noise in the data and in line with the recommendations for the use of mouse tracking [40,47,53], trials with response times longer than 5000 ms were excluded from the analysis (approximately 2% of the data).

### Statistical testing

Mixed effects-models [58–61] were used to assess the statistical significance of the effects, because these models are able to account for between-subject differences in participants’ basic motor properties, which are unrelated to the task design/experimental manipulation (e.g., a subject can show higher trajectories’ curvature or can be faster/slower compared to another, independently from the decision itself). Such approach represents a gold standard analysis when dealing with mouse kinematics data and was also employed in many previous mouse tracking studies [49–51,62–65].

The first set of analyses was conducted on the explicit measures and employed linear mixed-effect models (LMM) using the fixed-effect of condition (baseline, negative, neutral and positive) to predict different variables of the discounting behaviour. In the first analysis we tested whether the EFT manipulation was associated with a reduction of the individual discounting rates, and whether such a reduction was modulated by the emotional valence of the episodic tag. In the second analysis the same effect was tested on the distribution of choices for the two response types (i.e., now, later (see S1 File, S1 Fig). The third analysis, instead, which was conducted on the subjective values, was specifically aimed at testing whether the effect of the EFT manipulation was differential for choices associated with short- vs. long-time intervals (see also [46] for previous results from our group on this question). In this latter analysis, the original six time delays were collapsed into a short and a long time delay (i.e., short: 7, 15 and 30 days; long: 60, 90 and 180 days) and subjective values were predicted using an LMM with condition (baseline, negative, neutral and positive), time delay (short and long) and their interaction as fixed effects. The second set of analyses was conducted on kinematic data, using the different kinematics measures (i.e., MD, AUC, x-flips, total times, initiation times, motion times) as dependent variables, and the effect of condition (baseline, negative, neutral and positive), response type (now, later) and their interactions (condition by response type) as fixed effects.

A third set of analyses was finally aimed at testing whether the magnitude of the effect associated with the EFT/emotional valance manipulation was differential across individuals with different baseline discounting preferences (i.e., discounters vs. farsighted). More specifically, in line with the methodology employed in our previous studies [42,46], this analysis was conducted on both explicit and implicit measures and was performed by dividing our sample in discounters and farsighted individuals by means of a median split of the baseline discount rates (i.e., discounters: k > median; farsighted k < median, p < 0.001). In the first analysis, an index of the magnitude of the EFT manipulation for each emotional valence (i.e., *k shift*) was computed, which was derived from the difference between the baseline discount rate (*k*_*Base*_) and the discount rates associated with the three emotional valences obtained during the EFT session [i.e., *K shift*_*neg*_ *= K*_*base*_*−k*_*neg*_ *; K shift*_*neu*_ *= K*_*base*_*−k*_*neu*_*; K shift*_*pos*_ *= K*_*base*_*−k*_*pos*_]. As reported above, such an index was specifically intended to reflect the “size” of the discount rates’ modulation and to allow an estimate of the contribution of the baseline discounting preferences on the magnitude of the effect induced by the experimental manipulations. This could not be done by simply comparing discount rates of the two groups, since discounter’s and farsighted’s k-parameters are different by definition. The k shift index was used as dependent variable in a linear mixed-effect model with emotional valence (negative, neutral and positive), group (discounter, farsighted) and their interaction as fixed effects.

In the second analysis, we examined the same question on the pattern of the mouse trajectories associated with the choices by running a set of mixed-effect models using the kinematic measures (i.e., MD, AUC, x-flips, total times, initiation times, motion times) as dependent variables (i.e., separate models for each dependent variable) with condition (baseline, negative, neutral and positive), response type (now, later), group (discounter, farsighted) and their interactions as fixed effects.

All the mixed-effect models were conducted using the intercepts for *subjects*, and *trials* as random effects. All the post-hoc comparisons were run using a Tukey post-hoc analysis with FDR (i.e., False Rate Discovery) correction for multiple testing [66,67].

## Results

### Discount rate (k) modulation by EFT manipulation

**Fig 2:**
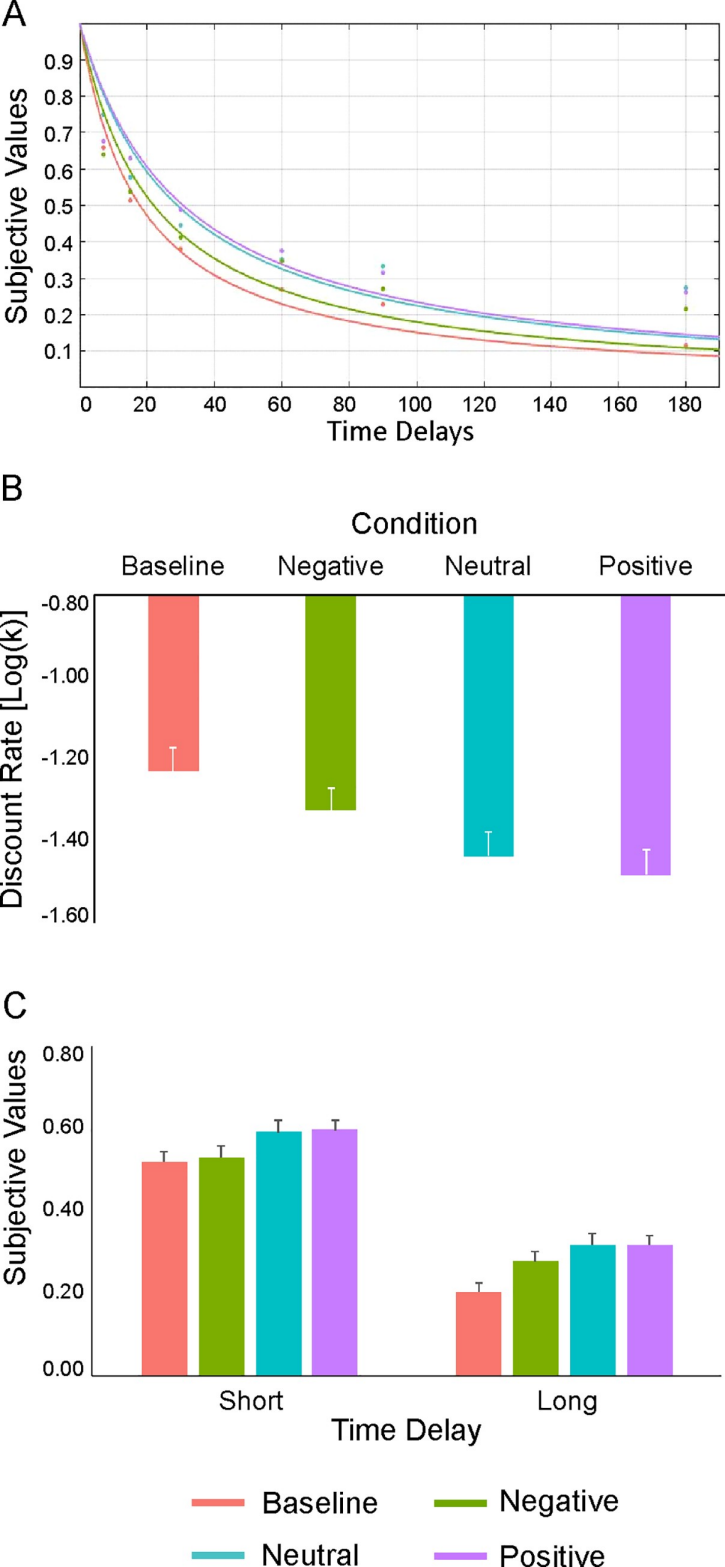
Effects of EFT manipulation on explicit measures. (A) Hyperbolic functions estimated across the four experimental conditions. (B) Results of the linear mixed-effects model conducted on discount rates (k) using the fixed effect of condition. (C) Results of the linear mixed-effects model conducted on subjective values using the fixed effect of condition, time delay and their interaction term.

The discounting functions (Fig 2A) and the discount rate (k; Fig 2B) range observed in our study across the four experimental conditions was in line with our previous studies [42,45,46] (baseline: m = -1.23 ± 0.416; negative: m = -1.33 ± 0.40; neutral: m = -1.44 ± 0.4; positive: m = -1.48 ± 0.46).

We next examined whether the EFT/emotional value manipulation was associated with a shift of the individual discounting preferences, and whether this effect was modulated by the emotional valence of the episodic tag, by running a linear mixed-effect model (LMM) with the condition as fixed effect. Because the condition factor included four levels (i.e., baseline, negative, neutral and positive), the baseline was considered as the default level of comparison. As displayed in Fig 2B, the results showed that the condition significantly predicted the discount rates: the baseline condition was associated with significantly higher discount rates as compared to the negative (β = -0.10, t = -34.27, p < 0.001), neutral (β = -0.21, t = -73.26, p < 0.001) and positive (β = -0.26, t = -91.00, p < 0.001) emotional valence conditions. Importantly, direct comparisons between the four levels revealed a significant difference between the three emotional valence conditions (Tukey post-hoc: k positive < k neutral < k negative, all p < 0.001) [66]. Therefore, the EFT manipulation was effective at reducing the individual discount rate and the modulation was mediated by a cumulative effect of concreteness and emotional valence.

Importantly, since the three emotional valence conditions were associated with different levels of arousal and relevance (see methods section), an additional series of LLM was computed in which the arousal and relevance rates were included as random effects. As reported in the results section of S1 File, a substantial replication of the original results was obtained, which confirmed that the observed reduction of the discount rates was generated by a cumulative effect of concreteness and emotional valence.

### Subjective values modulation by EFT manipulation

We next investigated whether the shift in discounting preferences produced by the three emotional valence conditions was guided by a modulation of subjective values (SV) at specific time delays (i.e., short vs. long). This analysis was motivated by our previous findings of a specific modulation of the discount rates by social modelling during short-, but not long-, time delays [46]. Hence, here we were interested in testing whether a manipulation specifically targeting the representation of the future (i.e., EFT) was able to shift even subjective values associated with longer time delays. To this aim, we conducted an LMM using the fixed effects of condition (default level: baseline), time delay (short, long; default level: long) and their interaction term. The results revealed a statistically significant effect of condition, indicating that the baseline was associated with significantly lower subjective values compared to the negative (β = 0.07, t = 19.03, p < 0.001), neutral (β = 0.11, t = 29.13, p < 0.001) and positive (β = 0.11, t = 29.24, p < 0.001) conditions (Fig 2C). Post-hoc inspection of the effect indicated that the negative condition was associated with significantly lower SV than the neutral and the positive conditions (negative < neutral; negative < positive, p < 0.001; neutral = positive, p = 0.86), thus paralleling the cumulative effect of concreteness and emotional valence observed on discount rates. As expected, the fixed effect of the time delay was also significant, with long time delays predicting overall lower subjective values than short time delays (β = 0.32, t = 80.40, p < 0.001). Finally, a statistically significant condition by time delay interaction was found (*Χ*^2^ = 137.88, p < 0.001), due to significant effects in all the post-hoc comparisons (p < 0.001), except for the difference between the neutral and positive conditions at short (p = 0.12) and long (p = 0.86) time delays.

As for the first analyses’ set on discount rates, the results were substantially replicated when controlling for relevance and arousal (see S1 File).

### Mouse kinematics modulations by EFT manipulation

**Fig 3:**
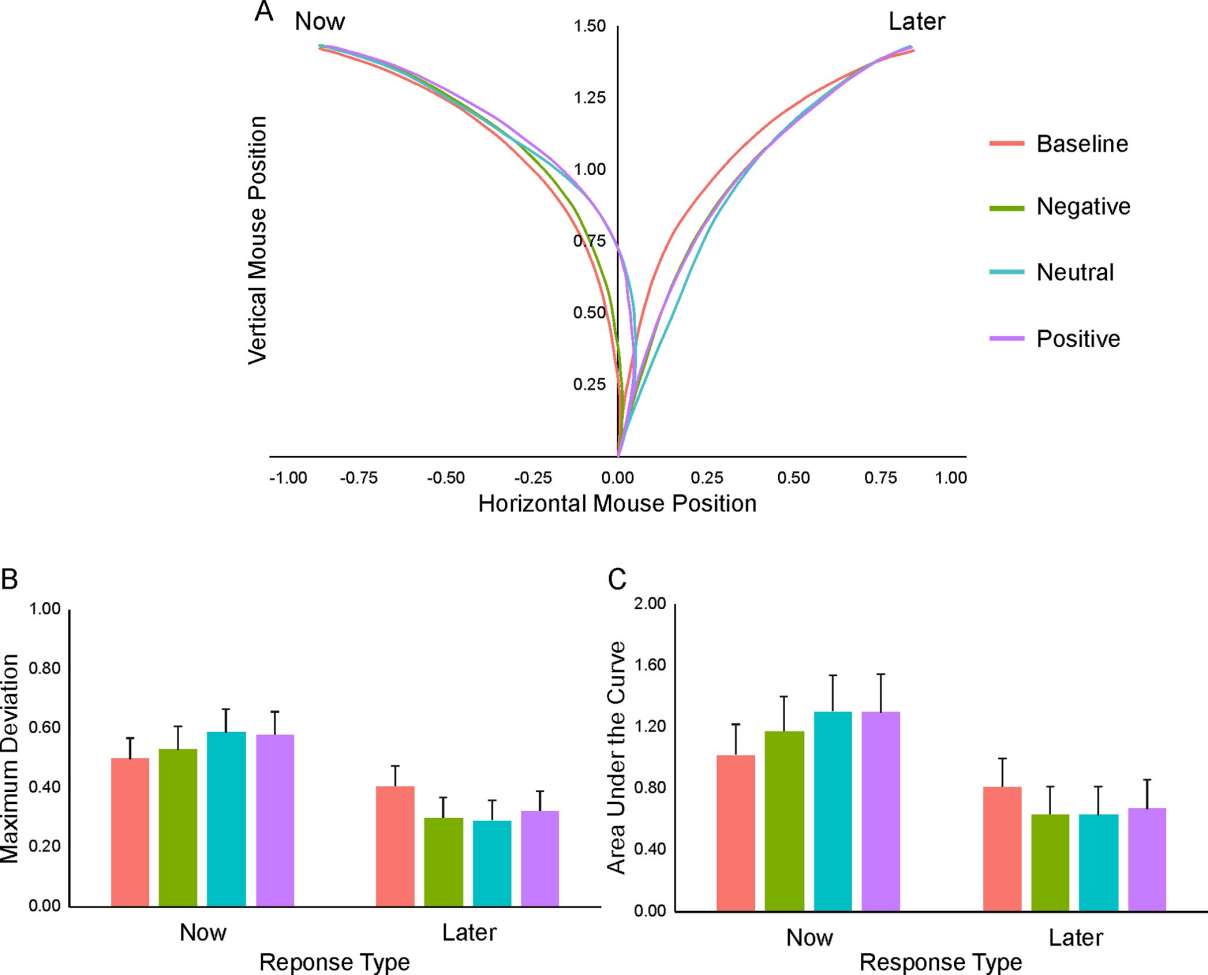
Effects of EFT manipulation on implicit measures. (A) Mean mouse trajectories across response type (now, later) and condition (baseline, negative, neutral and positive). (B, C) Results of the mixed-effects models conducted to predict maximum deviation (B) and area under the curve (C) using the fixed effects of the condition (baseline, negative, neutral and positive), response type (now, later) and the interaction term between them.

Besides choice outcome, we also examined the effect of the EFT manipulation on the intertemporal decision by extracting a series of kinematic measures providing an implicit index of choice uncertainty and changes of mind. Fig 3A displays the mean mouse trajectories across response type (now, later) and condition (baseline, negative, neutral and positive).

Analyses were conducted on both spatial (including *maximum deviation* or MD, *area under the curve* or AUC and *x-flips*) and temporal (including *total time*, *initiation time* and *motion time*) indices. Notably, however, since the analyses of the temporal indices and the x-flips might be affected by a task repetition/familiarity confound (i.e., shorter response latencies and less x-flips during the second EFT session vs. the first baseline session), here we specifically focused on MD and AUC measures (see Fig 3B and 3C, respectively). Based on their fine-graded sensitivity, moreover, these two measures are traditionally considered as the two most informative indicators of the mechanism of decision formation (i.e., unfolding of the decision process over time).

The statistical significance of the effects was tested using mixed-effects models with condition (baseline, negative, neutral and positive; default level of comparison: baseline), response type (now vs. later; default level: later) and their interaction term as fixed effects.

A statistically significant main effect of condition was found on both MD and AUC, which indicated a significantly higher curvature during baseline compared to the three EFT conditions (MD: *Χ*^*2*^ = 66.23, p < 0.001; AUC: *Χ*^*2*^ = 10.85, p < 0.01). The main effect of response type was also significant, indicating that the selection of the immediate vs. delayed alternative was associated with an overall more curved trajectory (MD: *Χ*^*2*^ = 1119.88, p < 0.001; AUC: *Χ*^*2*^ = 844.51, p < 0.001).

Importantly, a statistically significant condition by response type interaction was also found for both MD (*Χ*^*2*^ = 265.70, p < 0.001; Fig 3B) and AUC (*Χ*^*2*^ = 191.60, p < 0.001; Fig 3C), which was explained by an opposite pattern of attraction toward the unselected alternative in the three EFT conditions vs. baseline. As illustrated in Fig 3A, in particular, while the selection of the immediate option was associated with a progressive increase of attraction toward the (unselected) delayed option from the negative to the positive condition (MD and AUC: baseline < negative < neutral/positive, p < 0.001; neutral = positive, p > 0.05), the selection of the delayed option was associated with a specular decrease of attraction toward the immediate option (MD: baseline > negative/neutral/positive, p <0.001; negative = neutral, p = 0.07; positive > negative/neutral, p < 0.05; AUC: baseline > negative/neutral/positive, p < 0.001; negative = neutral/positive, p > 0.05; neutral < positive, p < 0.05) (see Table 1 for detailed results).

**Table 1:** Results of the linear mixed-effects models conducted on the spatial measures.

	Maximum Deviation			Area Under the Curve		
	*β*	*SE*	*t-value*	*β*	*SE*	*t-value*
*Intercept*	0.43	0.02	17.43***	0.85	0.06	14.38***
*Condition*: *Negative*	-0.11	0.01	-12.43***	-0.17	0.02	-6.98***
*Condition*: *Neutral*	-0.12	0.01	-14.31***	-0.20	0.02	-8.23***
*Condition*: *Positive*	-0.09	0.01	-10.72***	-0.16	0.02	-6.33***
*Response Type*: *Now*	0.05	0.01	5.22***	0.13	0.03	4.70***
*Condition*: *Negative* *Response Type*: *Now*	0.12	0.01	9.22***	0.30	0.04	7.77***
*Condition*: *Neutral* *Response Type*: *Now*	0.21	0.01	15.34***	0.50	0.04	12.65***
*Condition*: *Positive* *Response Type*: *Now*	0.17	0.01	12.19******	0.44	0.04	11.01***

The table shows the contrasts with the default level of comparison of each fixed-effect (condition: baseline; response type: later).

Statistical significance levels are indicated by the following symbol

*** p < 0.001.

It is worth noticing that a consistent pattern of results was obtained from the analyses of the other kinematic indices (see S1 File, S2 Fig and S1 Table). The same holds for the analyses controlling for arousal and relevance (i.e., ratings included in the LLM as random-effects), which were conducted on both spatial and temporal measures (see S1 File and S2 and S3 Tables).

### Effect of the baseline discounting preferences on the magnitude of the EFT modulation

After analysing the effect of the EFT manipulation on both explicit (response type, discount rates, subjective values) and implicit (spatial and temporal kinematics) measures, we investigated whether the effects were modulated by the individual variability in the baseline discounting preferences. In other words, we examined whether participants with different baseline preferences were equally susceptible to the EFT manipulation.

### Explicit measure (k shift)

Subjects were firstly divided in two groups based of their baseline discount rate (discounter vs. farsighted) and then an index of the discount rate modulation (or *k shift*) was computed for each subject. Finally, a LMM was conducted in order to predict the *k shift* using the emotional valence (default level: negative), the group (default level: discounter) and their interaction as fixed effects.

As reported in the methods section, this analysis was specifically conducted in order to test whether the extent to which participants were sensitive to the EFT manipulation was influenced by their individual preferences, and whether participants in the two groups were differentially affected by the emotional valence of the episodic tag.

**Fig 4:**
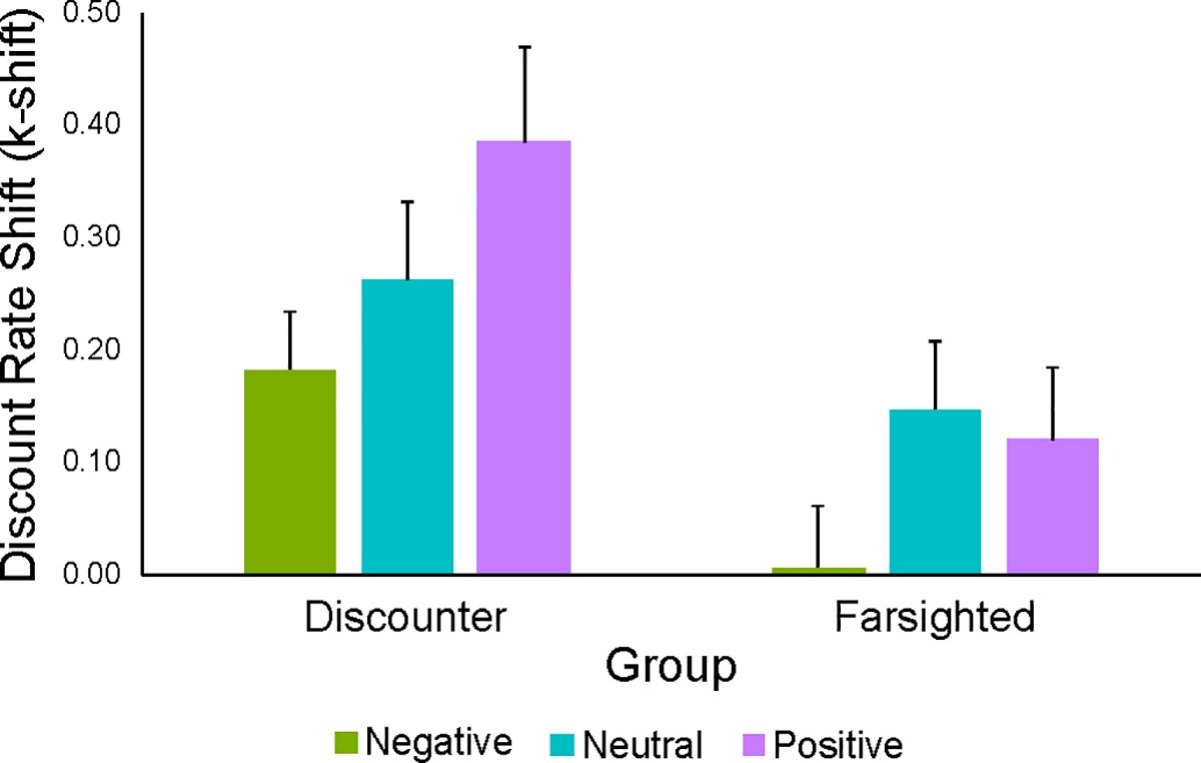
Influence of baseline preferences on explicit change measure (k-shift). Results of the linear mixed-effects models conducted to predict the magnitude of the EFT effect (k shift) using the fixed effect of the emotional valence (negative, neutral and positive), the group (discounter, farsighted) and the interaction term.

In line with the results reported in the previous sections, the emotional valence significantly predicted the magnitude of the *k shift* (*X*^*2*^ = 4161.48, p < 0.001), with negative emotional valence associated with lower *k shift* compared to both neutral (β = 0.08, t = 22.27, p < 0.001) and positive (β = 0.20, t = 57.42, p < 0.001) conditions. Furthermore, the positive EFT condition was associated with a higher *k shift* compared to the neutral condition (Tukey post-hoc test: p < 0.001) [66]. The results of the LMM, however, also showed a statistically significant effect of group (*X*^*2*^ = 5.72, p < 0.05), indicating that the magnitude of the EFT modulation was higher in discounters compared to farsighted individuals (β = -0.18, t = -2.28, p < 0.05). Finally, a significant condition by group interaction was found (*X*^*2*^ = 905.30, p < 0.001), which was explained by a significant difference between farsighted and discounter subjects in the magnitude of the *k shift* during the neutral and the positive EFT conditions. In particular, while farsighted individuals were equally susceptible to both neutral and positive EFT conditions (p > 0.05), discounter subjects showed a significantly higher *k shift* in the positive compared to the neutral EFT condition (p < 0.001) (Fig 4).

Notably, the results of this analysis were also replicated when including the arousal and relevance rates in the LLM as random-effects (see S1 File).

### Implicit measures (mouse kinematics)

We finally investigated whether the baseline discounting preferences also modulated the magnitude of the EFT effect on implicit measures of mouse kinematics. In particular, we tested whether the pattern of attraction toward the unselected response option was differentially modulated by the emotional valence in the two groups.

**Fig 5:**
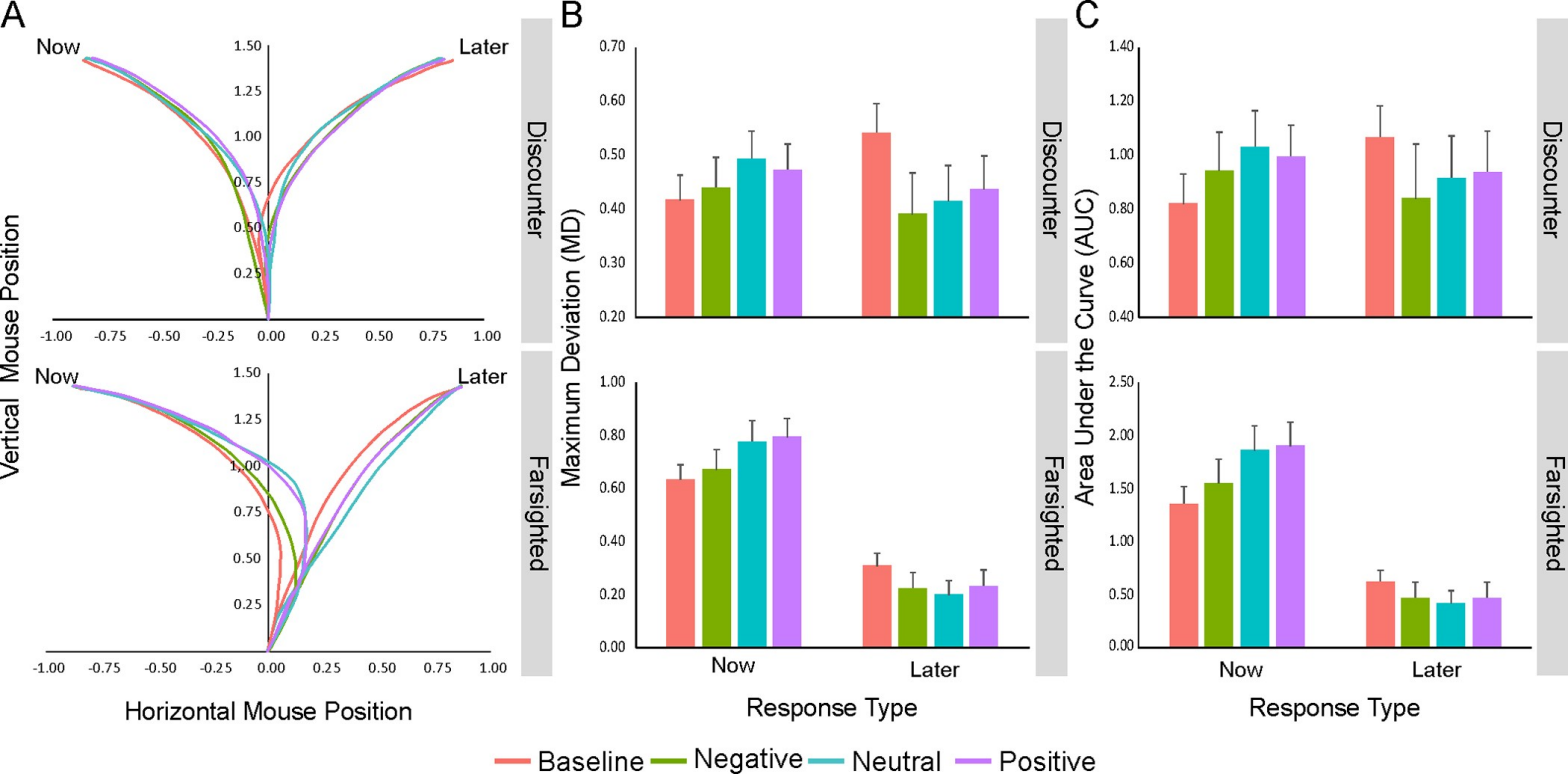
Influence of baseline preferences on the implicit measures (spatial indices of mouse kinematics). (**A**) Mean mouse trajectories associated with now/later responses in the two groups of discounters and farsighted participants and across the four experimental conditions. (B-C) Results of the linear mixed effect models conducted to predict the maximum deviation (B) and the area under the curve (C) using the fixed effects of the condition (baseline, negative, neutral and positive), response type (now, later), group (discounters, farsighted) and the second and third level interactions between them.

Fig 5A shows the mean mouse trajectories associated with now/later responses in the two groups of discounters and farsighted participants and across the four experimental conditions.

As for the kinematic analyses described above, mixed-effects models were performed on both MD and AUC using condition (default level: baseline), response type (default level: later), group (default level: discounters) and their interactions (second and third level) as fixed effects.

As reported in Table 2, several effects were found on these two measures [significant effect of condition (MD: *X*^*2*^ = 78.09, p < 0.001; AUC: *X*^*2*^ = 24.86, p < 0.001), response type (MD: *X*^*2*^ = 1167.42, p < 0.001; AUC: *X*^*2*^ = 1012.21, p < 0.001) and condition by response type interaction (MD: *X*^*2*^ = 313.90, p < 0.001; AUC: *X*^*2*^ = 191.56, p < 0.001); significant effect of group (MD: *X*^*2*^ = 0.90, p < 0.01; AUC: *X*^*2*^ = 8.80, p < 0.01), condition by group interaction (MD: *X*^*2*^ = 16.98, p < 0.001; AUC: *X*^*2*^ = 55.67, p < 0.001) and response by group interaction (MD: *X*^*2*^ = 2188.72, p < 0.001; AUC: *X*^*2*^ = 1411.39, p < 0.001)].

**Table 2:** Results of the linear mixed-effects models conducted to test the influence of baseline preferences on the spatial measures.

	Maximum Deviation (MD)			Area Under the Curve (AUC)		
	β	*SE*	*t value*	β	*SE*	*t value*
*Intercept*	0.55	0.03	16.23***	0.08	0.02	3.65***
*Condition*: *Negative*	-0.13	0.01	-10.21***	-0.11	0.03	-4.49***
*Condition*: *Neutral*	-0.13	0.01	-9.78***	-0.13	0.03	-5.01***
*Condition*: *Positive*	-0.11	0.01	-8.37***	-0.12	0.02	-4.72***
*Response*: *Now*	-0.15	0.01	-11.46***	-0.18	0.03	-7.11***
*Group*: *Farsighted*	-0.23	0.05	-4.73***	-0.28	0.03	-9.50***
*Condition*: *Negative* * *Response*: *Now*	0.12	0.02	6.83***	0.22	0.04	6.17***
*Condition*: *Neutral* * *Response*: *Now*	0.19	0.02	10.77***	0.26	0.04	7.27***
*Condition*: *Positive* * *Response*: *Now*	0.16	0.02	8.67***	0.23	0.04	6.46***
*Condition*: *Negative* * *Group*: *Farsighted*	0.04	0.02	2.15*	0.09	0.03	2.61**
*Condition*: *Neutral* * *Group*: *Farsighted*	0.01	0.02	0.39	0.10	0.03	3.13**
*Condition*: *Positive* * *Group*: *Farsighted*	0.03	0.02	1.51	0.11	0.03	3.41***
*Response*: *Now* * Group: Farsighted	0.44	0.02	23.04***	0.65	0.04	17.62***
*Condition*: *Negative* * *Response*: *Now* * Group: Farsighted	0.01	0.03	0.47	0.04	0.05	0.67
*Condition*: *Neutral* * *Response*: *Now* * Group: Farsighted	0.07	0.03	2.47*	0.19	0.05	3.61***
*Condition*: *Positive* * *Response*: *Now* * Group: Farsighted	0.06	0.03	2.35*	0.12	0.05	2.17*

The table shows the contrasts with the default level of comparison of each fixed-effect (condition: baseline; response type: later; group: discounter).

Statistical significance levels are indicated by the following symbols

*** p < 0.001

** p < 0.01

* p < 0.05.

However, of particular interest for the current issue was the third level interaction between condition, response type and group (MD: *X*^*2*^ = 9.72, p < 0.05; AUC: *X*^*2*^ = 15.46, p < 0.01; Fig 5B and 5C). This interaction reflected the different shape of the trajectories associated with the selection of the immediate vs. delayed alternative in the two groups across the experimental conditions. As shown in Fig 5A, in particular, the baseline kinematic pattern of farsighted participants was characterized by straight trajectories during the selection of the delayed option and by edge-curved trajectories during the selection of the immediate option (p < 0.001). Differently, the kinematic pattern of discounter participants was characterized by curved trajectories during the selection of both the immediate and the delayed option, with a more pronounced curvature when selecting the delayed one (p < 0.001). Therefore, the effect of the EFT manipulation on MD and AUC (i.e., increased attraction toward the delayed option when the immediate one was selected and, conversely, decreased attraction toward the immediate option when the delayed one was selected) was observed in both groups but was overall more pronounced in farsighted than in discounter participants. This was also reflected in the overall greater differences observed across the emotional valence conditions in farsighted compared to discounter participants. Consistent with our previous study [42], this difference may be due to the general baseline bias of farsighted individuals for the delayed option, which becomes also evident in their implicit behaviour.

Notably, a consistent pattern of results was observed in the analyses conducted on x-flips, total time, initiation time and motion time (see S1 File, S3 Fig and S4 Table) as well as in those controlling for arousal and relevance (see S1 File, S5 and S6 Tables).

## Discussion

A conspicuous amount of studies in the last few years have shown the attenuating effect of episodic future thinking on temporal discounting [25,31,32,35–38,68–73]. Despite the enthusiasm driven by the possibility to change individual preferences in discounting behaviour, however, the modulation elicited by episodic future thinking has also been discussed with some degree of scepticism. Indeed, it is not entirely clear how this effect works. Specifically, on the one hand, it has been proposed that this effect is a consequence of a change in the time construal associated with future time delays (i.e., from high level, abstract representations to low-level, concrete representations). On the other hand, since most of the previous works have used only positive tags, it has also been suggested that this modulation is guided by the positive emotions elicited by the episodic tags. Notably, moreover, even though some studies have tried to investigate this issue by introducing an emotional valence manipulation, the results are not yet conclusive, maybe due to the high inter-subject variability of the choice behaviour, which particularly affects between-subjects designs [35–38]. Furthermore, it should be noted that the procedure for EFT manipulation has not been always consistent across different studies. For example, while in some previous studies on episodic tagging participants were explicitly asked to imagine the future reward consumption in the circumstances signalled by the tag [31,71] or to imagine the future self immediately before the task [74], our procedure for the episodic tagging, which was based on the study by Peters & Büchel (2010) [25], did not involve an active/explicit mental reactivation of the tags. Additionally, with respect to the previous studies, here we manipulated the emotional valence of episodic future thinking using a within-subjects’ design in order to disentangle the contribution of concreteness and emotional valence to the observed effect of enhanced farsightedness induced by the EFT manipulation. To this aim we recorded both explicit (choice outcome) and implicit (mouse kinematics) measures. The results showed that: i) discount rates were significantly reduced in all the three emotional valence conditions with respect to baseline, with differences among them; ii) the effect of the EFT manipulation on subjective values was observed at both short and long time delays; iii) the effects were mediated by an increased attractiveness toward the delayed choice option and a decreased attractiveness toward the immediate one; iv) the effect of the EFT manipulation was differential in discounters vs. farsighted subjects (i.e., it was modulated by baseline discounting preferences) both at the level of the explicit and the implicit kinematic measures.

The finding (i) of the reduction of participants’ discount rates (k value) during the EFT session—in all the three emotional valences—as compared to baseline, supports the existence of a concreteness effect. Interestingly, however, the reduction was additionally modulated by the emotional valence of the episodic tag, with positive emotional valence condition associated with significantly greater modulations compared to neutral one, and neutral condition associated with greater modulations compared to negative one. On these bases, we conclude that the effect of enhanced farsightedness induced by our EFT manipulation is the product of the cumulative effect of concreteness and emotional valence. Accordingly, a recent study found that memory retrieval of positive personal events determined a reduction in discounting behaviour similar to that observed following the imagination/anticipation of positive future personal events [75]. The same did not happen for the retrieval (or future imagination) of positive events unrelated to personal memories (thus, less concrete), supporting the idea that emotional valence and concreteness independently influence discounting behaviour.

We next showed (ii) that the effect of increased farsightedness during the EFT manipulation was present at both short- and long-time delays. These findings are particularly relevant because they mark a difference with the results of our previous study on social modelling modulation of the discount rate, in which we found an effect for short time delays only [46]. This suggests that the EFT manipulation employed in the current study was able to determine a stronger change in the mental representation of future time delays, resulting in a more effective and “deep” manipulation. Taken together, these findings highlight that, in order to achieve a strong alteration of the subjective values during intertemporal decisions, it is essential to employ a manipulation that specifically targets the concreteness of future events, and in the context of this manipulation, it seems crucial to exploit the advantages offered by the positive emotional valence of the episodic tags.

As a third question, we investigated whether the modulation of the explicit choice measures was also apparent in implicit measures of choice selection. To this aim, we examined the mouse kinematics associated with the unfolding intertemporal decision. The kinematics results (iii) showed that the EFT manipulation produced the effect of increasing the attractiveness of the available future option as well as of reducing the attractiveness of the immediate one in all the three emotional valence conditions as compared to the baseline. Interestingly, moreover, the degree of this increased/decreased attraction was significantly modulated by the emotional valence of the episodic tag: mouse trajectories showed a “*gradient*” in the magnitude of the effect in both directions (i.e., increased/decreased attraction toward the delayed/immediate option) which was higher during the positive tags and progressively lower during the neutral and negative conditions. These results corroborate the idea that the increased farsightedness induced by the EFT manipulation is the aftermath of a cumulative effect of concreteness and emotional valence.

Finally, keeping in mind the relevance of these findings for possible applications to the clinical domain, we took a step forward by investigating whether the effect of the EFT manipulation on both explicit and implicit measures of choice selection was differential for discounters vs. farsighted subjects, or said in other words, whether the effect of the EFT was modulated by the baseline discounting preferences.

The results of these analyses (iv) showed that discounters exhibited an overall higher modulation of discount rates, which is probably due to the fact that these participants have more *“room for change”* as compared to the farsighted ones. Following the same idea but from the opposite side, one can also hypothesize that farsighted subjects were already so biased toward the delayed alternative at baseline that they could hardly additionally shift their choice pattern toward the delayed option. Therefore, the idea of a *“room for change”* proposed for the discounter subjects can be also framed in terms of a ceiling effect from the farsighted side. It should also be noted that these results are in line with those reported by Benoit and colleagues (2011), as the authors found that participants who—similarly to discounters in our study—are usually more indifferent toward long-term consequences of their actions (as tested using the CFC scale [76]), were more responsive to episodic prospection [71].

Besides a general effect of the baseline discounting preference on the k shift, we also found that negative cues were associated with a low magnitude of the discount rate shift across all participants, while neutral and positive tags had a different impact on discounters and farsighted individuals. Specifically, while no differential effect was observed for positive and negative tags in farsighted subjects, positive tags were more effective than neutral tags in discounter participants. Therefore, we hypothesize that in order to achieve a higher shift in k values, discounter participants need to take advantage of both the concreteness and positive emotional valence, while in farsighted subjects neutral and positive cues had the same impact on behaviour. This finding is particularly interesting for the clinical domain, as it suggests that when dealing with clinical populations characterized by higher-than-normal discount rates it is important to focus on both the affective and the prospection component in order to achieve a deeper change in behaviour.

Notably, the finding of a different discount rate modulation in discounters vs. farsighted subjects was further corroborated by the kinematics analysis, which showed that also the degree of attraction toward the immediate/delayed option was shaped by the baseline discounting preferences. Consistent with the results of our previous study [42], we observed that, in the baseline condition, while farsighted exhibited a clear motor bias toward the delayed option (i.e., straight trajectories associated with the selection of the delayed rewards and edge curved trajectories associated with the selection of the immediate one), discounter participants showed quite curved trajectories during the selection of both options. Importantly, in line with the baseline preferences (i.e., biases), the EFT modulations appeared to be overall more pronounced in farsighted than discounter participants. Such a finding highlights the relevance of including an implicit measure of the decision process in the experimental design. Indeed, while the explicit measures indicated a more pronounced effect in discounter than farsighted individuals, the implicit ones revealed a less pronounced increase in the attractiveness toward the delayed option in discounters than farsighted individuals. This is particularly relevant when dealing with clinical populations.

Overall, these results underline the importance of evaluating implicit mechanisms of decision behaviour, and changes in behaviour, as they may uncover hidden biases toward one of the alternatives, offering particularly interesting applications in the clinical domain. Indeed, the evaluation of the implicit dynamics underlying intertemporal decision-making may provide both an effective way of testing the efficacy of treatment/therapy and a possible predictive index of relapses, for example in presence of explicit changes in choice behaviour which are not (or not as much) paralleled by implicit changes in the attractiveness of the available options. Although intriguing, these aspects still need rigorous experimental investigation.

Before concluding, a limitation of the current study should be noted. Participants were not debriefed at the end of the experiment and, since they were not instructed to engage in an active/explicit mental reactivation of the episodic tags, we are not aware of the possible strategy they employed during the EFT session of the experiment.

## Conclusions

In line with relatively recent evolutionary considerations [77], the present results provide an additional piece of evidence in favour of the hypothesis that mental time travel and imaginative foresight may be crucial cognitive mechanisms for human decision making. In particular, according to this hypothesis, the ability to imagine and anticipate future events is thought to oppose the natural human tendency toward discounting, impulsive and opportunistic behaviour, nudging individuals towards choices which become advantageous in the long term [77]. Furthermore, the current implicit and explicit results also underlie the role of emotional valence in shifting individual discounting preferences, thus bringing support to integrated cognitive-affective models of intertemporal decision [14,15] and offering important insights into the clinical domain of reward-related disorders.

## Supporting information

S1 File Supporting methods and results. (DOCX)

S1 Fig Results of response type analyses. Results of the binomial mixed effect model conducted to predict response type (now, later) using the fixed effect of condition (baseline, negative, neutral, positive).
(TIF)

S2 Fig Results of the analysis on the additional spatial and temporal measures. Results of the mixed-effect models conducted using the fixed-effects of condition (baseline, negative, neutral and positive) and response type (now, later) on x-flips (A), total time (B), initiation time (C) and motion time (D).
(TIF)

S3 Fig Results of baseline modulations on the additional spatial and temporal measures. Results of the mixed-effect models conducted using the fixed-effects of group (discounters, farsighted) (i.e., based on the baseline preference), condition (baseline, negative, neutral and positive) and response type (now, later) on x-flips (A), total time (B), initiation time (C) and motion time (D).
(TIF)

S1 Table Results of the linear mixed-effect models conducted on the spatial and temporal measures. The table shows the contrasts with the default level of comparison of each fixed-effect (condition: baseline; response type: later). Statistical significance levels are indicated by the following symbols: *** p < 0.001; ** p < 0.01; * p < 0.05; Ϯ p < 0.1.
(DOCX)

S2 Table Results of the linear mixed-effect models conducted on the spatial measures controlled for arousal and relevance rate. The table shows the contrasts with the default level of comparison of each fixed-effect (condition: baseline; response type: later). Statistical significance levels are indicated by the following symbols: *** p < 0.001; ** p < 0.01; * p < 0.05.
(DOCX)

S3 Table Results of the linear mixed-effect models conducted on the temporal measures controlled for arousal and relevance rates. The table shows the contrasts with the default level of comparison of each fixed-effect (condition: baseline; response type: later). Statistical significance levels are indicated by the following symbols: *** p < 0.001; ** p < 0.01; Ϯ p < 0.1.
(DOCX)

S4 Table Results of the linear mixed-effect models conducted on the spatial and temporal measures to test the influence of baseline preferences. The table shows the contrasts with the default level of comparison of each fixed-effect (condition: baseline; response type: later; group: discounters). Statistical significance levels are indicated by the following symbols: *** p < 0.001; ** p < 0.01; * p < 0.05; Ϯ p < 0.1.
(DOCX)

S5 Table Results of the linear mixed-effect models conducted on the spatial measures to test the influence of baseline preferences controlling for arousal and relevance rates. The table shows the contrasts with the default level of comparison of each fixed-effect (condition: baseline; response type: later; group: discounters). Statistical significance levels are indicated by the following symbols: *** p < 0.001; ** p < 0.01; * p < 0.05; Ϯ p < 0.1.
(DOCX)

S6 Table Results of the linear mixed-effect models conducted on the temporal measures to test the influence of baseline preferences controlling for arousal and relevance rates. The table shows the contrasts with the default level of comparison of each fixed-effect (condition: baseline; response type: later; group: discounters). Statistical significance levels are indicated by the following symbols: *** p < 0.001; ** p < 0.01; * p < 0.05.
(DOCX)
