# Supplementary material for: Concreteness and emotional valence of episodic future thinking (EFT) independently affect the dynamics of intertemporal decisions
Source: PLoS One. 2019 May 28;14(5):e0217224. doi: 10.1371/journal.pone.0217224 (PMC6538244; doi:10.1371/journal.pone.0217224)
Supplement: S1 File — (DOCX) [file pone.0217224.s001.docx]

# **Supplementary information:**

# **Emotional valence of Episodic Future Thinking (EFT) affects the dynamics of intertemporal decisions**

Calluso C.^1,2*^, Tosoni A.^1^, Cannito L.^1^, Committeri G.^1^

^1^Department of Neuroscience, Imaging and Clinical Sciences, Gabriele d’Annunzio University, and Institute for Advanced Biomedical Technologies (ITAB), Chieti scalo, 66013, Italy.

^2^ Department of Business and Management, LUISS Guido Carli University, Rome, 00197, Italy.

**Corresponding Author**:

*Cinzia Calluso

Department of Business and Management

LUISS Guido Carli University

Viale Romania, 32 - 00197

Roma, Italy

e-mail address: [ccalluso@luiss.it](mailto:ccalluso@luiss.it)

# **Methods and Results**

## **Response type modulations by EFT manipulation**

In addition to the discount rates (k) modulation by the EFT manipulation (see main text), the effect of the EFT emotional valence was also tested on the distribution of choices for the immediate vs. the delayed alternative. To this aim, a binomial mixed-effect model was employed to predict the response type (i.e., now vs. later) using the fixed effect of condition (baseline, negative, neutral and positive; default level of comparison: baseline). The intercepts for subjects, trials, personal relevance rates and arousal rates were included as random effects. The results showed that the response type was significantly predicted by the condition (S1 Fig). Specifically, a significantly higher number of delayed choices was found in the negative (β = -0.38, z = -13.29, p < 0.001), neutral (β = -0.51, z = -17.38, p < 0.001) and positive (β = -0.57, z = -19.57, p < 0.001) conditions compared to the baseline. Importantly, moreover, direct comparisons between the four levels indicated a significant difference between the three EFT conditions, thus indicating that the number of later responses significantly increased from the negative to the neutral and the positive emotional valence condition (Tukey post-hoc test FDR corrected for multiple testing; response type later: baseline < negative < neutral < positive p < 0.01). Based on these findings, we concluded that the observed increase in the percentage of later vs. now responses in the EFT session was mediated by a cumulative effect of concreteness (i.e., EFT sessions vs. baseline) and emotional valence (i.e., positive vs. neutral vs. negative emotional valence condition).

## **Response type, discount rates and subjective values analyses controlled for relevance and arousal rates**

All the analyses conducted on the explicit measures described in the main text and in the previous section (i.e., response type) were run also controlling for relevance rate and arousal rate. Specifically, a binomial mixed-effect model was run on the response type (i.e., now, later), using the fixed effect of condition (baseline, negative, neutral, positive). Similarly, a linear mixed-effect model (LMM) was run to predict the individuals’ discount rates (k) using the fixed-effect of condition (baseline, negative, neutral and positive). Finally, an LLM was conducted to predict subjective values using a LMM with the fixed effect of condition (baseline, negative, neutral and positive), time delay (short and long) and the interaction term between them. Thus, the original six time delays were collapsed into two intervals of short (7, 15 and 30 days) and long (60, 90 and 180 days) time delays. Crucially, all the mixed-effect models described above were conducted using the intercepts for subjects, trials, personal relevance rate and arousal rate as random effects. These latter were included as random effects in order ensure that the results were not affected by the differences in relevance and arousal rates among the episodic tags.

The results obtained on the response type replicated those reported above: a statistically significant higher number of delayed choices was observed in the negative (β = -0.59, z = -6.53, p < 0.001), neutral (β = -0.62, z = -7.29, p < 0.001) and positive (β = -0.80, z = -8.65, p < 0.001) condition as compared to the baseline session (Tukey post-hoc test FDR corrected for multiple testing; response type later: baseline < negative < neutral < positive p < 0.01; negative = neutral, p > 0.05).

Similarly, discount rates associated with the negative (β = -0.06, t = -8.18, p < 0.001), neutral (β = -0.17, t = -23.16, p < 0.001) and positive (β = -0.23, t = -27.80, p < 0.001) condition were lower as compared to the baseline session even when controlling for relevance and arousal rates. Further, the direct comparisons between the levels indicated that the three emotional valences all differed among each other (Tukey post-hoc: k positive < k neutral < k negative, all p < 0.00).

Finally, the analysis conducted on the subjective values by controlling for relevance and arausal rate revealed a statistically significant effect of condition (*X^2^* = 453.04, p <0.001), indicating that the baseline was associated with significantly lower subjective values compared to the neutral (β = 0.04, t = 5.17, p < 0.001) and positive (β = 0.05, t = 6.15, p < 0.001) conditions, but the baseline did not differed from the negative condition (β = 0.01, t = 1.18, p = 0.24). The fixed effect of the time delay was also significant, with long time delays predicting overall lower subjective values than short time delays (β = 0.34, t = 88.12, p < 0.001). Finally, a statistically significant condition by time delay interaction was found (*Χ^2^* = 271.24, p < 0.001), due to significant effects in all the post-hoc comparisons (p < 0.001), except for the difference between the baseline and negative conditions at long (p = 0.25) time delays, and the difference between the baseline and the positive condition at short time delay (p = 0.36).

## **Mouse kinematics modulations by EFT manipulation and control for relevance and arousal rates**

As indicated in the main text of the manuscript, the same analyses reported for maximum deviation (MD) and area under the curve (AUC) were also conducted on the other spatial measure (i.e., x-flips: number of times the mouse cursor changes direction along the x-axis) and on all temporal measures (i.e., total time; the time between the trial onset and the response selection; initiation time: the time between the trial onset and the movement’s initiation; motion time: the time between the movement’ initiation and the response selection). To this aim, linear models were used for the analyses of the temporal measures while a Poisson model was used to predict the number of x-flips. The models were run separately for each dependent variable using condition (baseline, negative, neutral and positive; default level of comparison: baseline), response type (now vs. later; default level: later) and their interaction as fixed effects, while the intercepts for *subjects* and *trials* were included as random effects.

The detailed results of these analyses are reported in S1 Table. A statistically significant effect of the condition was found in all the dependent variables, thus suggesting that the baseline was associated with a significantly higher hesitation (x-flips: *Χ^2^* = 391.31, p < 0.001) and latency (total time: *Χ^2^* = 2941.32, p < 0.001; initiation time: *Χ^2^* = 1074.91, p < 0.001; motion time: *Χ^2^* = 1724.46, p < 0.001) compared to the negative, neutral and positive emotional valence conditions. Further, the main effect of response type was also significant, indicating that the selection of the immediate vs. the delayed alternative was associated with overall slower trajectories (total time: *Χ^2^* = 777.22, p < 0.001; initiation time: *Χ^2^* = 166.55, p < 0.001; motion time: *Χ^2^* = 540.24, p < 0.001), but not with higher hesitation (x-flips: p = 0.11). Finally, a statistically significant condition by response type interaction was also observed for the x-flips (*Χ^2^* = 19.55, p < 0.001) and for all the temporal measures (total time: *Χ^2^* = 105.17, p < 0.001; initiation time: *Χ^2^* = 9.18, p < 0.05; motion time: *Χ^2^* = 97.04, p < 0.001) but, differently from the MD and AUC parameters (see main text, Table 1, Figure 3), it is possible that the large difference observed between the baseline and the EFT session (see S2 Fig) may have flattened the differences across the three emotional valences in the EFT session.

Moreover, all the analyses conducted on the kinematic measures were controlled for the different level of arousal and relevance between the three emotional valence conditions by including the arousal and relevance rates in the model as random effects (i.e., random effect structure: intercepts for subjects, trials, personal relevance rate and arousal rate; see S2 and S3 Tables).

## **Effect of baseline discounting preferences and control for relevance and arousal rates**

As reported in the main text, a series of analyses were conducted in order to investigate whether the effect of the EFT/emotional valence manipulation was differential across individual with different baseline discounting preferences (i.e., discounters vs. farsighted). As listed below, the results of these analyses are replicated when controlling for arousal and relevance (intercepts for subject, trials, arousal rate and relevance rate included in the model as random effects). Specifically, the results indicated that the emotional valence significantly predicted the magnitude of the *k shift* (*X^2^* = 4221.39, p < 0.001), with negative emotional valence associated with lower *k shift* compared to both neutral (β = 0.07, t = 18.43, p < 0.001) and positive (β = 0.20, t = 57.34, p < 0.001) conditions. Furthermore, the positive EFT condition was associated with a higher *k shift* compared to the neutral condition (Tukey post-hoc test: p < 0.001). The results of the LMM, however, also showed a statistically significant effect of group (*X^2^* = 5.84 p < 0.05), indicating that the magnitude of the EFT modulation was higher in discounters compared to farsighted individuals (β = -0.18, t = -2.32, p < 0.05). Finally, a significant condition by group interaction was found (*X^2^* = 865.81, p < 0.001), which was explained by a significant difference between farsighted and discounter subjects in the magnitude of the *k shift* during the neutral and the positive EFT conditions. In particular, while farsighted individuals were equally susceptible to both neutral and positive EFT conditions (p > 0.05), discounter subjects showed a significantly higher *k shift* in the positive compared to the neutral EFT condition (p < 0.001).

Finally, the effect of the baseline discounting preferences on the magnitude of the EFT/emotional valence modulation was also tested on x-flips and temporal measures of mouse kinematics by using mixed-effect models with condition (baseline, negative, neutral and positive; default level: baseline), response type (now and later, default level: later), group (discounters and farsighted; default level: discounters) and their interaction (second and third level) as fixed effects (intercepts for *subjects* and *trials* included as random effects). Linear models were used to predict the total time, initiation time and motion time, while a Poisson model was employed to predict x-flips.

The detailed results of these analyses are reported in S4 Table (see S3 Fig). A statistically significant effect of condition (x-flips: *X^2^* = 388.98, p < 0.001; total time: *X^2^* = 2949.19, p < 0.001; initiation time: *X^2^* = 1075.15, p < 0.001; motion time: *X^2^* = 1727.06, p < 0.001), response type (x-flips: *X^2^* = 77.55, p < 0.001; total time: *X^2^* = 779.07, p < 0.001; initiation time: *X^2^* = 163.22, p < 0.001; motion time: *X^2^* = 544.21, p < 0.001), and condition by response type interaction (x-flips: *X^2^* = 17.91, p < 0.001; total time: *X^2^* = 93.89, p < 0.001; initiation time: *X^2^* = 7.45, p < 0.05; motion time: *X^2^* = 91.85, p < 0.001) was observed in all the dependent variables, replicating the results obtained in the analyses reported above (section 1.2.).

A significant effect of group was also observed on the x-flips (*X^2^* = 4.06, p < 0.05) but not for any of the temporal measures (total time: *X^2^* = 0.61, p = 0.43; initiation time: *X^2^* = 0.00, p = 0.99; motion time: *X^2^* = 0.67, p = 0.41). The condition by group interaction did not reach the statistical significance in any of the dependent variables (x-flips: *X^2^* = 3.78, p = 0.29; total time: *X^2^* = 12.88, p < 0.01; initiation time: *X^2^* = 32.57, p < 0.001; motion time: *X^2^* = 2.46, p =0.48). Conversely, a statistically significant response by group interaction was observed in all the kinematic measures (x-flips: *X^2^* = 60.02, p < 0.001; total time: *X^2^* = 289.43, p < 0.001; initiation time: *X^2^* = 0.56, p = 0.45; motion time: *X^2^* = 317.58, p < 0.001). Finally, the third level interaction (i.e., condition by response by group) did not reach the statistical significance neither for the x-flips parameter nor for the other temporal measures (x-flips: *X^2^* = 0.91, p = 0.82; total time: *X^2^* = 5.57, p = 0.13; initiation time: *X^2^* = 2.68, p = 0.44; motion time: *X^2^* = 5.12, p = 0.16).

As shown for previous results, the analyses controlling for arousal and relevance rates replicated the original findings (see S5 and S6 Tables).
