# Supplementary figures and images for: Concreteness and emotional valence of episodic future thinking (EFT) independently affect the dynamics of intertemporal decisions
Source: PLoS One. 2019 May 28;14(5):e0217224. doi: 10.1371/journal.pone.0217224 (PMC6538244; doi:10.1371/journal.pone.0217224)

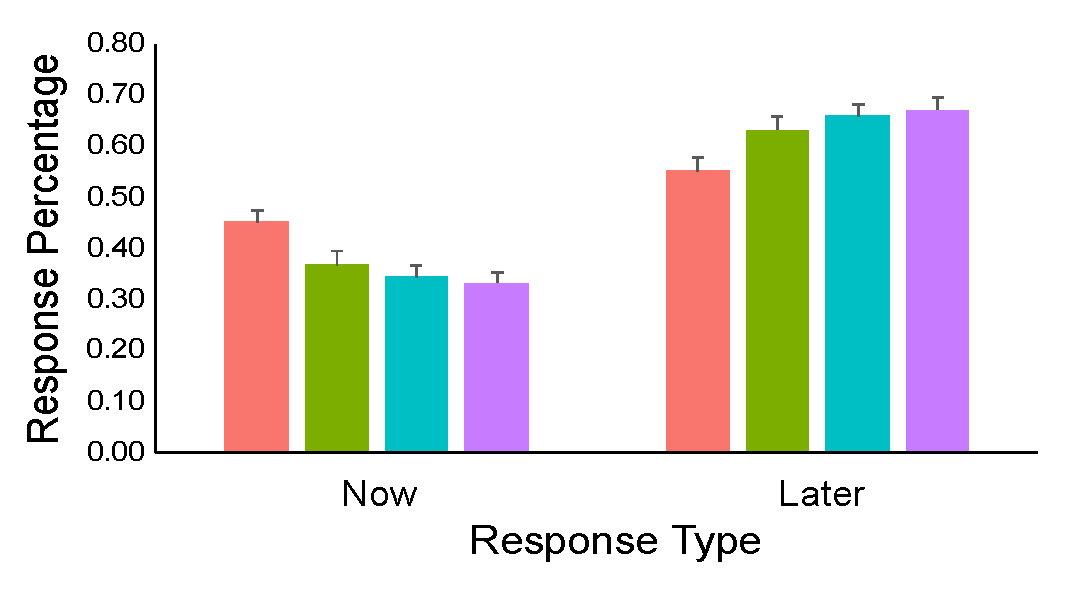

Supplement: S1 Fig — Results of the binomial mixed effect model conducted to predict response type (now, later) using the fixed effect of condition (baseline, negative, neutral, positive). (TIF) [file pone.0217224.s002.tif]

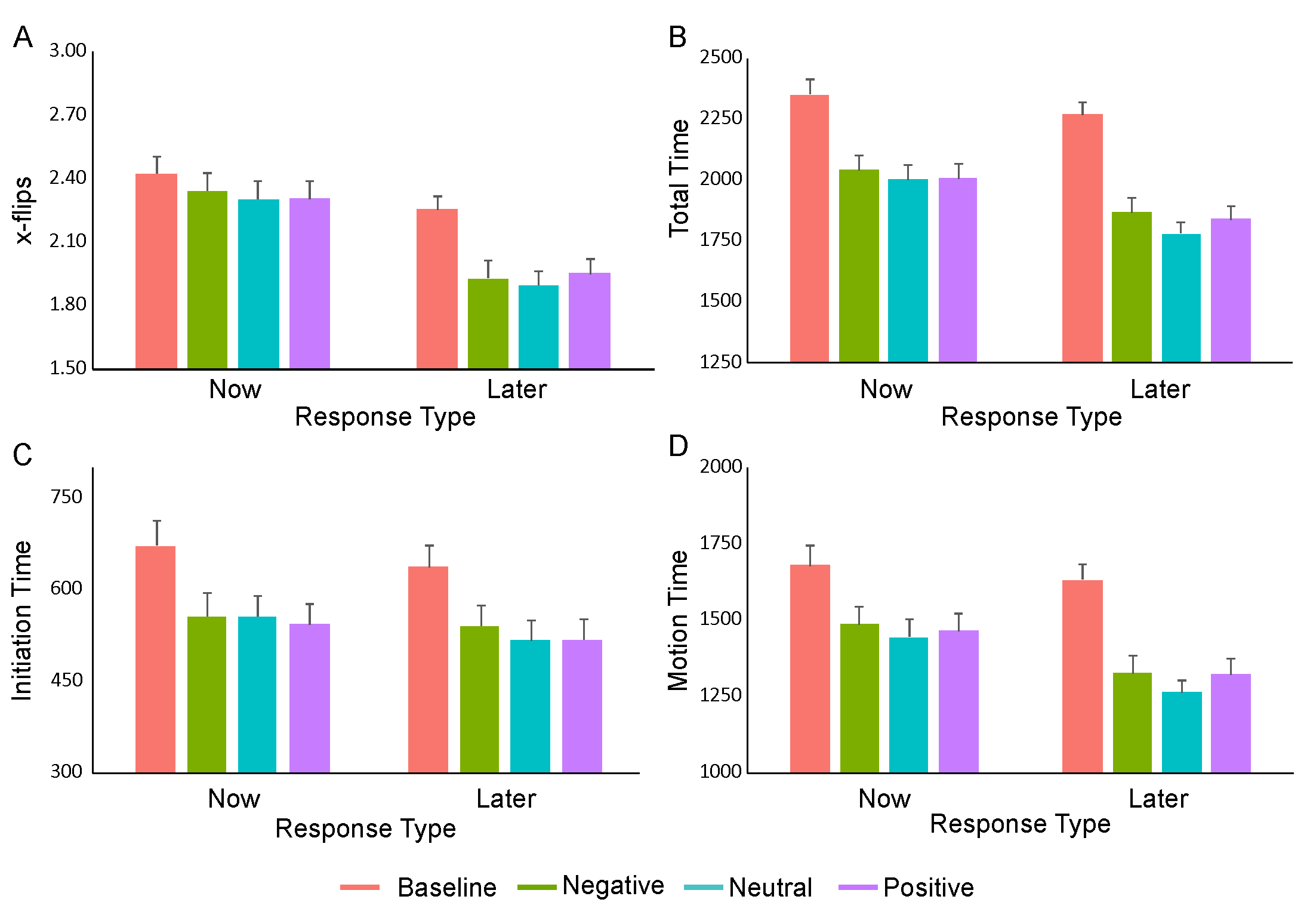

Supplement: S2 Fig — Results of the mixed-effect models conducted using the fixed-effects of condition (baseline, negative, neutral and positive) and response type (now, later) on x-flips (A), total time (B), initiation time (C) and motion time (D). (TIF) [file pone.0217224.s003.tif]

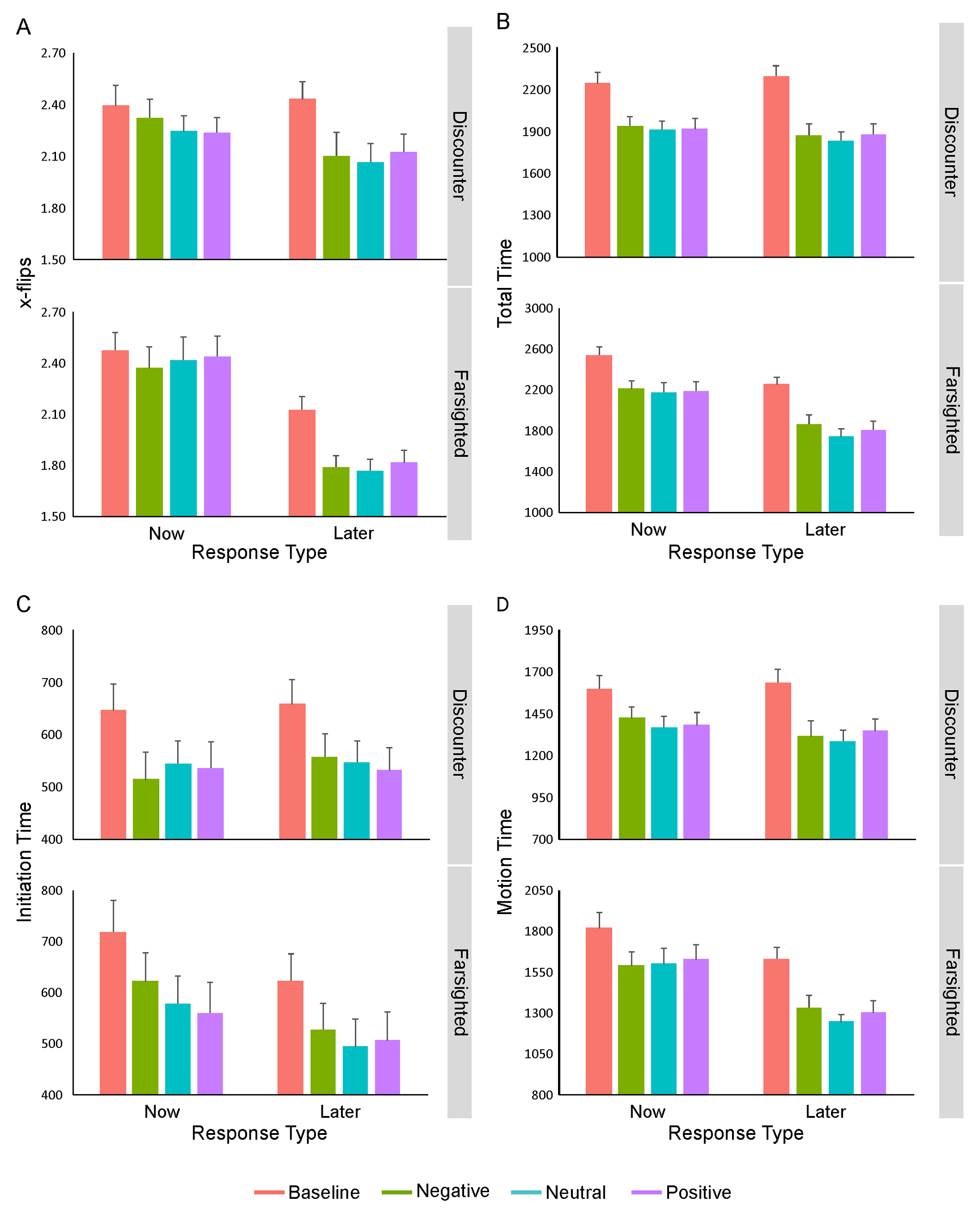

Supplement: S3 Fig — Results of the mixed-effect models conducted using the fixed-effects of group (discounters, farsighted) (i.e., based on the baseline preference), condition (baseline, negative, neutral and positive) and response type (now, later) on x-flips (A), total time (B), initiation time (C) and motion time (D). (TIF) [file pone.0217224.s004.tif]
