# Supplementary material for: Concreteness and emotional valence of episodic future thinking (EFT) independently affect the dynamics of intertemporal decisions
Source: PLoS One. 2019 May 28;14(5):e0217224. doi: 10.1371/journal.pone.0217224 (PMC6538244; doi:10.1371/journal.pone.0217224)
Supplement: S1 Table — The table shows the contrasts with the default level of comparison of each fixed-effect (condition: baseline; response type: later). Statistical significance levels are indicated by the following symbols: *** p < 0.001; ** p < 0.01; * p < 0.05; Ϯ p < 0.1. (DOCX) [file pone.0217224.s005.docx]

**S1 Table. Results of the linear mixed-effect models conducted on the spatial and temporal measures.**

|  | x-flips | | |  | Total Time | | |  | Initiation Time | | |  | Motion Time | | |
| --- | --- | --- | --- | --- | --- | --- | --- | --- | --- | --- | --- | --- | --- | --- | --- |
|  | *β* | *SE* | *z-value* |  | *β* | *SE* | *t-value* |  | *β* | *SE* | *t-value* |  | *β* | *SE* | *t-value* |
| *Intercept* | 0.76 | 0.03 | 27.58^***^ |  | 2273.14 | 46.47 | 48.92^***^ |  | 637.86 | 32.81 | 19.44^***^ |  | 1635.15 | 43.32 | 37.74^***^ |
| *Condition: Negative* | -0.15 | 0.01 | -12.51^***^ |  | -412.54 | 11.7 | -35.25^***^ |  | -107.06 | 5.54 | -19.32^***^ |  | -305.37 | 10.96 | -27.87^***^ |
| *Condition: Neutral* | -0.2 | 0.01 | -16.38^***^ |  | -507.87 | 11.57 | -43.89^***^ |  | -123.95 | 5.48 | -22.62^***^ |  | -383.87 | 10.83 | -35.44^***^ |
| *Condition: Positive* | -0.16 | 0.01 | -13.30^***^ |  | -440 | 11.54 | -38.14^***^ |  | -130.48 | 5.46 | -23.89^***^ |  | -309.51 | 10.8 | -28.66^***^ |
| *Response Type: Now* | 0.02 | 0.01 | 1.61 |  | 90.19 | 13.01 | 6.93^***^ |  | 29.25 | 6.16 | 4.75^***^ |  | 60.93 | 12.18 | 5.01^***^ |
| *Condition: Negative Response Type: Now* | 0.05 | 0.02 | 2.67^**^ |  | 122.74 | 18.41 | 6.67^***^ |  | 12.41 | 8.72 | 1.42 |  | 109.96 | 17.23 | 6.38^***^ |
| *Condition: Neutral Response Type: Now* | 0.08 | 0.02 | 4.33^***^ |  | 183.66 | 18.48 | 9.94^***^ |  | 16.71 | 8.75 | 1.91^Ϯ^ |  | 166.96 | 17.3 | 9.65^***^ |
| *Condition: Positive Response Type: Now* | 0.05 | 0.02 | 2.54^*^ |  | 121.68 | 18.6 | 6.54^***^ |  | 26.19 | 8.81 | 2.97^**^ |  | 95.49 | 17.41 | 5.49^***^ |

The table shows the contrasts with the default level of comparison of each fixed-effect (condition: baseline; response type: later). Statistical significance levels are indicated by the following symbols: *** p < 0.001; ** p < 0.01; * p < 0.05; Ϯ p < 0.1.
