# Supplementary material for: Concreteness and emotional valence of episodic future thinking (EFT) independently affect the dynamics of intertemporal decisions
Source: PLoS One. 2019 May 28;14(5):e0217224. doi: 10.1371/journal.pone.0217224 (PMC6538244; doi:10.1371/journal.pone.0217224)
Supplement: S2 Table — The table shows the contrasts with the default level of comparison of each fixed-effect (condition: baseline; response type: later). Statistical significance levels are indicated by the following symbols: *** p < 0.001; ** p < 0.01; * p < 0.05. (DOCX) [file pone.0217224.s006.docx]

**S2 Table. Results of the linear mixed-effect models conducted on the spatial measures controlled for arousal and relevance rate.**

|  | Maximum Deviation | | |  | Area Under the Curve | | |  | x-flips | | |
| --- | --- | --- | --- | --- | --- | --- | --- | --- | --- | --- | --- |
|  | *β* | *SE* | *t-value* |  | *β* | *SE* | *t-value* |  | *β* | *SE* | *z-value* |
| *Intercept* | 0.40 | 0.03 | 14.24*** |  | -0.10 | 0.03 | -4.00** |  | 0.76 | 0.03 | 27.58*** |
| *Condition: Negative* | -0.08 | 0.01 | -5.46*** |  | -0.03 | 0.03 | -1.26 |  | -0.15 | 0.01 | -12.51*** |
| *Condition: Neutral* | -0.09 | 0.01 | -6.39*** |  | -0.04 | 0.03 | -1.52 |  | -0.20 | 0.01 | -16.38*** |
| *Condition: Positive* | -0.06 | 0.01 | -4.35*** |  | -0.03 | 0.03 | -1.12 |  | -0.16 | 0.01 | -13.30*** |
| *Response Type: Now* | 0.05 | 0.01 | 5.20*** |  | 0.12 | 0.02 | 6.61*** |  | 0.02 | 0.01 | 1.61 |
| *Condition: Negative Response Type: Now* | 0.12 | 0.01 | 9.18*** |  | 0.22 | 0.03 | 8.28*** |  | 0.05 | 0.02 | 2.67** |
| *Condition: Neutral Response Type: Now* | 0.21 | 0.01 | 15.32*** |  | 0.28 | 0.03 | 10.82*** |  | 0.08 | 0.02 | 4.33*** |
| *Condition: Positive Response Type: Now* | 0.17 | 0.01 | 12.16*** |  | 0.23 | 0.03 | 8.74*** |  | 0.05 | 0.02 | 2.54* |

The table shows the contrasts with the default level of comparison of each fixed-effect (condition: baseline; response type: later). Statistical significance levels are indicated by the following symbols: *** p < 0.001; ** p < 0.01; * p < 0.05.
