# Supplementary material for: Concreteness and emotional valence of episodic future thinking (EFT) independently affect the dynamics of intertemporal decisions
Source: PLoS One. 2019 May 28;14(5):e0217224. doi: 10.1371/journal.pone.0217224 (PMC6538244; doi:10.1371/journal.pone.0217224)
Supplement: S3 Table — The table shows the contrasts with the default level of comparison of each fixed-effect (condition: baseline; response type: later). Statistical significance levels are indicated by the following symbols: *** p < 0.001; ** p < 0.01; Ϯ p < 0.1. (DOCX) [file pone.0217224.s007.docx]

**S3 Table. Results of the linear mixed-effect models conducted on the temporal measures controlling for arousal and relevance rates.**

|  | Total time | | |  | Initiation Time | | |  | Motion Time | | |
| --- | --- | --- | --- | --- | --- | --- | --- | --- | --- | --- | --- |
|  | *β* | *SE* | *t-value* |  | *β* | *SE* | *t-value* |  | *β* | *SE* | *t-value* |
| *Intercept* | 2359.20 | 59.29 | 39.79*** |  | 676.76 | 35.74 | 18.94*** |  | 1673.67 | 50.62 | 33.07*** |
| *Condition: Negative* | -521.56 | 25.84 | -20.19*** |  | -149.83 | 11.81 | -12.69*** |  | -360.71 | 23.31 | -15.47*** |
| *Condition: Neutral* | -610.45 | 24.48 | -24.94*** |  | -172.08 | 11.16 | -15.42*** |  | -427.52 | 22.12 | -19.32*** |
| *Condition: Positive* | -553.65 | 26.48 | -20.91*** |  | -171.61 | 12.08 | -14.21*** |  | -371.26 | 23.90 | -15.54*** |
| *Response Type: Now* | 90.30 | 13.00 | 6.95*** |  | 29.20 | 6.16 | 4.74*** |  | 61.10 | 12.17 | 5.02*** |
| *Condition: Negative Response Type: Now* | 121.39 | 18.41 | 6.60*** |  | 12.71 | 8.72 | 1.46 |  | 108.46 | 17.23 | 6.29*** |
| *Condition: Neutral Response Type: Now* | 181.76 | 18.49 | 9.83*** |  | 14.81 | 8.76 | 1.69 Ϯ |  | 166.97 | 17.31 | 9.65*** |
| *Condition: Positive Response Type: Now* | 122.85 | 18.60 | 6.60*** |  | 27.56 | 8.81 | 3.13** |  | 95.18 | 17.42 | 5.47*** |

The table shows the contrasts with the default level of comparison of each fixed-effect (condition: baseline; response type: later). Statistical significance levels are indicated by the following symbols: *** p < 0.001; ** p < 0.01; Ϯ p < 0.1.
