# Supplementary material for: Concreteness and emotional valence of episodic future thinking (EFT) independently affect the dynamics of intertemporal decisions
Source: PLoS One. 2019 May 28;14(5):e0217224. doi: 10.1371/journal.pone.0217224 (PMC6538244; doi:10.1371/journal.pone.0217224)
Supplement: S4 Table — The table shows the contrasts with the default level of comparison of each fixed-effect (condition: baseline; response type: later; group: discounters). Statistical significance levels are indicated by the following symbols: *** p < 0.001; ** p < 0.01; * p < 0.05; Ϯ p < 0.1. (DOCX) [file pone.0217224.s008.docx]

**S4 Table**. **Results of the linear mixed-effect models conducted on the spatial and temporal measures.**

|  | Total Time | | |  | Initiation Time | | |  | Motion Time | | |  | x-flips | | |
| --- | --- | --- | --- | --- | --- | --- | --- | --- | --- | --- | --- | --- | --- | --- | --- |
|  | *β* | *SE* | *t-value* |  | *β* | *SE* | *t-value* |  | *β* | *SE* | *t-value* |  | *β* | *SE* | *z-value* |
| *Intercept* | 2271.71 | 63.26 | 35.91*** |  | 639.11 | 46.31 | 13.80*** |  | 1632.59 | 60.15 | 27.14*** |  | 0.82 | 0.04 | 21.63*** |
| *Condition: Negative* | -411.79 | 17.78 | -23.16*** |  | -122.33 | 8.45 | -14.48*** |  | -289.50 | 16.64 | -17.40*** |  | -0.13 | 0.02 | -7.40*** |
| *Condition: Neutral* | -463.77 | 17.78 | -26.08*** |  | -108.27 | 8.45 | -12.82*** |  | -355.55 | 16.64 | -21.36*** |  | -0.19 | 0.02 | -10.13*** |
| *Condition: Positive* | -418.42 | 17.64 | -23.73*** |  | -129.25 | 8.38 | -15.43*** |  | -289.02 | 16.50 | -17.51*** |  | -0.14 | 0.02 | -7.74*** |
| *Response: Now* | 0.33 | 17.93 | 0.02 |  | 23.13 | 8.52 | 2.72** |  | -22.90 | 16.78 | -1.37 |  | -0.02 | 0.02 | -0.87 |
| *Group: Farsighted* | 11.61 | 87.50 | 0.13 |  | -2.27 | 65.76 | -0.03 |  | 13.66 | 84.12 | 0.16 |  | -0.12 | 0.05 | -2.22* |
| *Condition: Negative * Response: Now* | 112.68 | 25.28 | 4.46*** |  | 24.91 | 12.00 | 2.08* |  | 87.71 | 23.66 | 3.71*** |  | 0.04 | 0.03 | 1.39 |
| *Condition: Neutral * Response: Now* | 144.19 | 25.17 | 5.73*** |  | 10.77 | 11.95 | 0.90 |  | 133.50 | 23.55 | 5.67*** |  | 0.07 | 0.03 | 2.59** |
| *Condition: Positive * Response: Now* | 117.94 | 25.22 | 4.68*** |  | 33.57 | 11.98 | 2.80** |  | 83.88 | 23.60 | 3.55*** |  | 0.03 | 0.03 | 1.37 |
| *Condition: Negative * Group: Farsighted* | -2.70 | 23.56 | -0.12 |  | 27.49 | 11.19 | 2.46* |  | -29.90 | 22.05 | -1.36 |  | -0.04 | 0.02 | -1.52 |
| *Condition: Neutral * Group: Farsighted* | -75.90 | 23.36 | -3.25** |  | -27.22 | 11.10 | -2.45* |  | -48.51 | 21.86 | -2.22* |  | -0.03 | 0.02 | -1.18 |
| *Condition: Positive * Group: Farsighted* | -37.93 | 23.26 | -1.63 |  | -2.15 | 11.05 | -0.20 |  | -36.01 | 21.76 | -1.66Ϯ |  | -0.04 | 0.02 | -1.73Ϯ |
| *Response: Now * Group: Farsighted* | 217.44 | 26.47 | 8.21*** |  | 15.83 | 12.57 | 1.26 |  | 201.77 | 24.77 | 8.15*** |  | 0.10 | 0.03 | 3.61*** |
| *Condition: Negative * Response: Now * Group: Farsighted* | 18.07 | 37.55 | 0.48 |  | -20.66 | 17.83 | -1.16 |  | 38.08 | 35.14 | 1.08 |  | 0.02 | 0.04 | 0.55 |
| *Condition: Neutral * Response: Now * Group: Farsighted* | 77.10 | 38.20 | 2.02* |  | -0.97 | 18.14 | -0.05 |  | 78.00 | 35.74 | 2.18* |  | 0.04 | 0.04 | 0.94 |
| *Condition: Positive * Response: Now * Group: Farsighted* | -4.44 | 38.39 | -0.12 |  | -22.46 | 18.23 | -1.23 |  | 19.33 | 35.93 | 0.54 |  | 0.02 | 0.04 | 0.49 |

The table shows the contrasts with the default level of comparison of each fixed-effect (condition: baseline; response type: later; group: discounters). Statistical significance levels are indicated by the following symbols: *** p < 0.001; ** p < 0.01; * p < 0.05; Ϯ p < 0.1.
