# Supplementary material for: Concreteness and emotional valence of episodic future thinking (EFT) independently affect the dynamics of intertemporal decisions
Source: PLoS One. 2019 May 28;14(5):e0217224. doi: 10.1371/journal.pone.0217224 (PMC6538244; doi:10.1371/journal.pone.0217224)
Supplement: S5 Table — The table shows the contrasts with the default level of comparison of each fixed-effect (condition: baseline; response type: later; group: discounters). Statistical significance levels are indicated by the following symbols: *** p < 0.001; ** p < 0.01; * p < 0.05; Ϯ p < 0.1. (DOCX) [file pone.0217224.s009.docx]

**S5 Table**. **Results of the linear mixed-effect models conducted on the spatial measures controlling for arousal and relevance rates.**

|  | Maximum Deviation | | |  | Area Under the Curve | | |  | x-flips | | |
| --- | --- | --- | --- | --- | --- | --- | --- | --- | --- | --- | --- |
|  | *β* | *SE* | *t-value* |  | *β* | *SE* | *t-value* |  | *β* | *SE* | *z-value* |
| *Intercept* | 0.53 | 0.04 | 14.50*** |  | 0.07 | 0.03 | 2.29* |  | 0.82 | 0.04 | 21.63*** |
| *Condition: Negative* | -0.11 | 0.02 | -6.55*** |  | -0.11 | 0.03 | -3.18** |  | -0.13 | 0.02 | -7.40*** |
| *Condition: Neutral* | -0.09 | 0.02 | -5.58*** |  | -0.11 | 0.03 | -3.30** |  | -0.19 | 0.02 | -10.13*** |
| *Condition: Positive* | -0.09 | 0.02 | -5.13*** |  | -0.12 | 0.03 | -3.38** |  | -0.14 | 0.02 | -7.74*** |
| *Response: Now* | -0.15 | 0.01 | -11.47*** |  | -0.18 | 0.03 | -7.09*** |  | -0.02 | 0.02 | -0.87 |
| *Group: Farsighted* | -0.23 | 0.05 | -4.72*** |  | -0.28 | 0.03 | -9.43*** |  | -0.12 | 0.05 | -2.22* |
| *Condition: Negative * Response: Now* | 0.12 | 0.02 | 6.81*** |  | 0.22 | 0.04 | 6.14*** |  | 0.04 | 0.03 | 1.39 |
| *Condition: Neutral * Response: Now* | 0.19 | 0.02 | 10.70*** |  | 0.26 | 0.04 | 7.27*** |  | 0.07 | 0.03 | 2.59** |
| *Condition: Positive * Response: Now* | 0.16 | 0.02 | 8.62*** |  | 0.23 | 0.04 | 6.42*** |  | 0.03 | 0.03 | 1.37 |
| *Condition: Negative * Group: Farsighted* | 0.04 | 0.02 | 2.19* |  | 0.09 | 0.03 | 2.68** |  | -0.04 | 0.02 | -1.52 |
| *Condition: Neutral * Group: Farsighted* | 0.00 | 0.02 | 0.17 |  | 0.10 | 0.03 | 3.10** |  | -0.03 | 0.02 | -1.18 |
| *Condition: Positive * Group: Farsighted* | 0.03 | 0.02 | 1.51 |  | 0.11 | 0.03 | 3.40*** |  | -0.04 | 0.02 | -1.73 Ϯ |
| *Response: Now * Group: Farsighted* | 0.44 | 0.02 | 23.08*** |  | 0.66 | 0.04 | 17.65** |  | 0.10 | 0.03 | 3.62*** |
| *Condition: Negative * Response: Now * Group: Farsighted* | 0.01 | 0.03 | 0.41 |  | 0.03 | 0.05 | 0.64 |  | 0.02 | 0.04 | 0.55 |
| *Condition: Neutral * Response: Now * Group: Farsighted* | 0.07 | 0.03 | 2.49* |  | 0.20 | 0.05 | 3.66*** |  | 0.04 | 0.04 | 0.94 |
| *Condition: Positive * Response: Now * Group: Farsighted* | 0.06 | 0.03 | 2.34* |  | 0.12 | 0.05 | 2.18* |  | 0.02 | 0.04 | 0.49 |

The table shows the contrasts with the default level of comparison of each fixed-effect (condition: baseline; response type: later; group: discounters). Statistical significance levels are indicated by the following symbols: *** p < 0.001; ** p < 0.01; * p < 0.05; Ϯ p < 0.1.
