# Supplementary material for: Concreteness and emotional valence of episodic future thinking (EFT) independently affect the dynamics of intertemporal decisions
Source: PLoS One. 2019 May 28;14(5):e0217224. doi: 10.1371/journal.pone.0217224 (PMC6538244; doi:10.1371/journal.pone.0217224)
Supplement: S6 Table — The table shows the contrasts with the default level of comparison of each fixed-effect (condition: baseline; response type: later; group: discounters). Statistical significance levels are indicated by the following symbols: *** p < 0.001; ** p < 0.01; * p < 0.05. (DOCX) [file pone.0217224.s010.docx]

**S6 Table**. **Results of the linear mixed-effect models conducted on the spatial measures controlling for arousal and relevance rates.**

|  | Total Time | | |  | Initiation Time | | |  | Motion Time | | |
| --- | --- | --- | --- | --- | --- | --- | --- | --- | --- | --- | --- |
|  | *β* | *SE* | *t-value* |  | *β* | *SE* | *t-value* |  | *β* | *SE* | *z-value* |
| *Intercept* | 2361.59 | 73.74 | 32.03*** |  | 678.10 | 48.50 | 13.98*** |  | 1675.66 | 65.84 | 25.45*** |
| *Condition: Negative* | -526.76 | 28.80 | -18.29*** |  | -165.12 | 13.29 | -12.43*** |  | -351.69 | 26.25 | -13.40*** |
| *Condition: Neutral* | -564.42 | 27.81 | -20.30*** |  | -155.97 | 12.76 | -12.23*** |  | -398.84 | 25.42 | -15.69*** |
| *Condition: Positive* | -541.26 | 29.69 | -18.23*** |  | -171.43 | 13.66 | -12.55*** |  | -359.83 | 27.05 | -13.30*** |
| *Response: Now* | 0.51 | 17.93 | 0.03 |  | 23.10 | 8.52 | 2.71** |  | -22.54 | 16.78 | -1.34 |
| *Group: Farsighted* | 11.22 | 87.80 | 0.13 |  | -2.40 | 65.91 | -0.04 |  | 13.56 | 84.20 | 0.16 |
| *Condition: Negative * Response: Now* | 112.56 | 25.30 | 4.45*** |  | 25.75 | 12.01 | 2.14* |  | 86.60 | 23.68 | 3.66*** |
| *Condition: Neutral * Response: Now* | 140.33 | 25.19 | 5.57*** |  | 9.18 | 11.96 | 0.77 |  | 131.03 | 23.57 | 5.56*** |
| *Condition: Positive * Response: Now* | 119.41 | 25.23 | 4.73*** |  | 35.45 | 11.98 | 2.96** |  | 83.25 | 23.62 | 3.53*** |
| *Condition: Negative * Group: Farsighted* | -4.66 | 23.57 | -0.20 |  | 26.34 | 11.20 | 2.35* |  | -30.86 | 22.06 | -1.40 |
| *Condition: Neutral * Group: Farsighted* | -85.55 | 23.45 | -3.65*** |  | -28.09 | 11.13 | -2.52* |  | -57.04 | 21.95 | -2.60** |
| *Condition: Positive * Group: Farsighted* | -36.15 | 23.24 | -1.56 |  | -1.78 | 11.04 | -0.16 |  | -34.81 | 21.76 | -1.60 |
| *Response: Now * Group: Farsighted* | 218.63 | 26.47 | 8.26*** |  | 16.10 | 12.57 | 1.28 |  | 202.34 | 24.77 | 8.17*** |
| *Condition: Negative * Response: Now * Group: Farsighted* | 12.89 | 37.60 | 0.34 |  | -22.76 | 17.85 | -1.28 |  | 35.74 | 35.20 | 1.02 |
| *Condition: Neutral * Response: Now * Group: Farsighted* | 76.84 | 38.21 | 2.01* |  | -2.40 | 18.15 | -0.13 |  | 79.92 | 35.77 | 2.23* |
| *Condition: Positive * Response: Now * Group: Farsighted* | -4.52 | 38.41 | -0.12 |  | -23.64 | 18.24 | -1.30 |  | 20.60 | 35.95 | 0.57 |

The table shows the contrasts with the default level of comparison of each fixed-effect (condition: baseline; response type: later; group: discounters). Statistical significance levels are indicated by the following symbols: *** p < 0.001; ** p < 0.01; * p < 0.05.
